# Neural Mechanisms of Learning and Consolidation of Morphologically Derived Words in a Novel Language: Evidence From Hebrew Speakers

**DOI:** 10.1162/nol_a_00150

**Published:** 2024-09-11

**Authors:** Upasana Nathaniel, Stav Eidelsztein, Kate Girsh Geskin, Brianna L. Yamasaki, Bracha Nir, Vedran Dronjic, James R. Booth, Tali Bitan

**Affiliations:** Institute of Information Processing and Decision Making, University of Haifa, Haifa, Israel; Department of Communication Sciences and Disorder, University of Haifa, Haifa, Israel; Department of Psychology, Emory University, Atlanta, GA, USA; Department of English, Northern Arizona University, Flagstaff, AZ, USA; Department of Psychology and Human Development, Vanderbilt University, Nashville, TN, USA; Department of Speech Pathology, University of Toronto, Toronto, Ontario, Canada

**Keywords:** artificial language, decomposition, derivational, fMRI, morphology, second language acquisition

## Abstract

We examined neural mechanisms associated with the learning of novel morphologically derived words in native Hebrew speakers within the Complementary Learning Systems (CLS) framework. Across four sessions, 28 participants were trained on an artificial language, which included two types of morphologically complex words: linear (root + suffix) with a salient structure, and non-linear (root interleaved with template), with a prominent derivational structure in participants’ first language (L1). A third simple monomorphemic condition, which served as baseline, was also included. On the first and fourth sessions, training was followed by testing in an fMRI scanner. Our behavioural results showed decomposition of both types of complex words, with the linear structure more easily learned than the non-linear structure. Our fMRI results showed involvement of frontal areas, associated with decomposition, only for the non-linear condition, after just the first session. We also observed training-related increases in activation in temporal areas specifically for the non-linear condition, which was correlated with participants’ L1 morphological awareness. These results demonstrate that morphological decomposition of derived words occurs in the very early stages of word learning, is influenced by L1 experience, and can facilitate word learning. However, in contrast to the CLS framework, we found no support for a shift from reliance on hippocampus to reliance on cortical areas in any of our conditions. Instead, our findings align more closely with recent theories showing a positive correlation between changes in hippocampus and cortical areas, suggesting that these representations co-exist and continue to interact with one another beyond initial learning.

## INTRODUCTION

Learning a second language (L2) as an adult is often challenging. One of the hardest aspects of late L2 learning is learning grammar (Johnson & Newport, 1989; Ullman, 2004), particularly the processing and production of L2 morphology (Bowden et al., 2010; Clahsen et al., 2010; McCarthy, 2008). The current study examined the neural processes underlying morphological decomposition, that is, the ability to segment words into smaller meaningful units, or morphemes (e.g., *un-learn-able*), in the early stages of learning a new language. In light of the Complementary Learning Systems (CLS) theory (McClelland et al., 1995) and its adaptation to language learning (Davis & Gaskell, 2009), the study also examined the role of consolidation processes and their underlying neural mechanisms in learning to decompose morphologically derived words. This was tested by using functional magnetic resonance imaging (fMRI) to examine Hebrew speaking participants before and after multisession training on a new morpholexicon. This study was preregistered.

### Learning and Consolidation of Novel Words

The neural basis of learning novel words has recently been explained within the CLS framework. The CLS model was first developed to provide a computational basis of learning new information from experience (McClelland et al., 1995). The model addresses the differential contributions of hippocampal and neocortical structures to memory. Based on this model, while initial encoding of new information is said to be dependent on the hippocampal system via episodic memories, later long-term retention of information is said to be dependent on neocortical structures via semantic memories, with strengthening of one system and weakening of the dependence on the other occurring during offline consolidation. A similar trajectory has been proposed when learning new words (Davis & Gaskell, 2009), such that novel words are initially hippocampus dependent, and following a period of consolidation are integrated within the existing lexicon in neocortical structures, forming long-term semantic representations (Dumay & Gaskell, 2007; Tamminen et al., 2010).

More recently, evidence from declarative memory research questions the purported shift from hippocampal to neocortical involvement and suggests a more gradual and continued involvement of the hippocampus in later stages of learning (Gilboa & Moscovitch, 2021; Sekeres et al., 2018; Winocur et al., 2010; Winocur & Moscovitch, 2011). Neuroimaging studies of vocabulary learning have shown that early hippocampal activation during encoding stages correlates with successful learning on the same day of training (Breitenstein et al., 2005; Davis et al., 2009), as well as correlations between hippocampal volume and L2 proficiency after 3 months of language study (Mårtensson et al., 2012). While there is no direct evidence for reduced hippocampal involvement in later stages of L2 learning, several studies have shown an increase in activation in neocortical regions such as the inferior frontal gyrus (IFG), superior temporal gyrus (STG), posterior middle temporal gyrus (MTG), and inferior parietal lobule in L2 vocabulary learning, on the same day of exposure to novel words (Veroude et al., 2010), after 6 weeks (Yang et al., 2015), and after 16 weeks of training (Hosoda et al., 2013).

Previous studies have also established the key role of consolidation in the integration and retention of new linguistic knowledge (Dumay & Gaskell, 2007). During consolidation, repeated aspects are extracted from episodic traces, thus strengthening the learning of overlapping information and inducing generalization (Lewis & Durrant, 2011). The ability to generalize or extract general information from individual learning episodes is fundamental to language learning and is especially relevant to learning grammar, in particular morphological regularities. Few studies have examined learning and consolidation of new morphologically derived words (Leminen et al., 2016; Tamminen et al., 2012; Tamminen et al., 2015). The studies that have been conducted have investigated the integration of newly learnt words into an existing lexicon by combining existing words with new morphemes (e.g., *sleep-nule*), and demonstrating successful generalization of novel affixes (i.e., *nule*) to untrained words (e.g., *sail-nule*) after a 24-hour period of consolidation. The current study aims to extend this work by examining the role of consolidation and associated neural processes in the learning of completely novel words, with and without internal morphological structure.

### Neural Basis of Processing Morphological Derivations

Many languages construct word forms by concatenating a morphological affix to a stem or base; for instance, the inflectional suffix -*s* may indicate plurality in English (as in “flowers”), and the derivational suffix -*er* can indicate a doer or agent (as in “gardener” = a person who gardens). While inflectional processes yield semantic variations of the same lexeme (e.g., “walk” and “walked”), derivational affixes add semantic information that yields distinct lexemes (e.g., “walk” and “walker”) and can change word category. Few behavioural studies have examined morphological decomposition, specifically when learning a second language. While some have shown comparable morphological priming effects for derived words in native (L1) and L2 speakers, suggesting that words were decomposed into their constituents in both groups of speakers (Diependaele et al., 2011; Jacob et al., 2018; Kirkici & Clahsen, 2013; Voga et al., 2014), others have shown reduced sensitivity or no priming effects for morphologically related pairs in L2 compared to L1 speakers, suggesting holistic processing of derived words in L2 (Clahsen et al., 2010; Fernandes et al., 2023; Silva & Clahsen, 2008).

In contrast to the consistent findings for inflectional morphology, showing an association of decomposition with left IFG (LIFG) pars opercularis (Bulut, 2022; Desai et al., 2006; Pliatsikas, Johnstone, & Marinis, 2014; Sahin et al., 2006; Tyler et al., 2005), neuroimaging studies of derivational morphology in L1 show mixed results regarding evidence for decomposition (Leminen et al., 2019). While some studies show evidence for increased activity in LIFG (Bozic et al., 2007) supporting decompositional processes, others show increased bilateral frontotemporal activations of derived forms (Klimovich-Gray et al., 2017; Meinzer et al., 2009) which may suggest whole-word processing (Bozic et al., 2013). With regard to decomposition in L2 morphology, a recent passive listening electroencephalography study, using real Finnish stems combined with existing derivational, inflectional, and pseudo-suffixes, demonstrated early decomposition of inflections and derivations in advanced L2 learners, while beginner learners of L2 relied more on full-form processing of derivations (Kimppa et al., 2019). The study further suggested that neural differences between L1 and L2 speakers appeared to decrease as L2 proficiency increased. An magnetoencephalography (MEG) study examining the role of consolidation in learning derivational morphology, using novel affixes with existing words, showed stronger activity in LIFG for trained novel words compared to untrained words, suggesting decomposition immediately after exposure to the novel morphology, while differences in STG appeared following overnight consolidation (Leminen et al., 2016). However, these studies relied on existing vocabulary with novel affixes, making it easier to identify and decompose the new morphemes. The current study focused on the neural basis of decomposition in learners of a novel language, using derivations that do not share morphemes with existing words.

### Impact of Morphological Mechanisms on Decomposition

Studies of morphological learning in a new language suggest that prior morphological knowledge in the native language plays an important role in the learning process (Havas et al., 2015; Luk & Shirai, 2009; Murakami & Alexopoulou, 2016). Several studies have demonstrated that learners of new languages show higher sensitivity to morphological structures if these structures were also present in their native language (Drake, 2018; LaCross, 2015; Norman et al., 2016). Studies of morphological processing have typically focused on linear morphology, which is characterized by a sequential concatenation of prefixes or suffixes to a root morpheme, such as in *bright-ness* (Gor & Jackson, 2013; Li et al., 2017; Meunier & Longtin, 2007). Words can also be structured non-linearly, for example, by interweaving a consonantal root and a vocalic (vowel-based) template. This morphological mechanism is characteristic of Semitic languages such as Hebrew, the native language of participants in the current study. Many of the words in Hebrew are constructed by interweaving the phonological constituents of a root morpheme, which consist of (typically) three discontinuous consonants, with a vocalic template consisting of a sequence of non-adjacent vowels (Shimron, 2003). For example, the Hebrew word *xizúk* (meaning “strengthening”), is composed non-linearly by combining the root morpheme x-z-k (general meaning of “force”) with the template *CiCúC* (C = consonant), which is one of the Hebrew patterns used for deriving nominalizations. The non-linear structure serves as a major mechanism of derivational formation in Hebrew (Bolozky, 2007). It is the only way to derive verbs and is a prevalent mechanism in deriving nouns (Schwarzwald, 2002). Behavioural studies have demonstrated that the discontinuous consonantal root morpheme is activated during lexical access (Frost et al., 1997; Frost et al., 2000; Velan et al., 2005), and acts as an organizing unit in the mental lexicon of Hebrew speakers (Berman & Seroussi, 2011; Frost et al., 2005).

While non-linear derivation is very common in Hebrew, linear morphology is also a commonly used derivational mechanism, especially in the nominal domain (Schwarzwald, 2006). For example, the word *pardes-an* (meaning “orange grove keeper”), is derived from the noun *pardes* (meaning “orange grove”) and the agent suffix -*an*. Moreover, non-linearly derived words can be recursively combined with linear concatenations to derive new words such as *nigud-i-ut* (opposition_n_ – > opposite_adj_ – > oppositeness_n_). Because linear derivations are constructed using distinct units, which in Hebrew often undergo fewer changes when assembled into new words (Schwarzwald, 2002), it is possible that learning of linear structure may be easier in comparison to the non-linear structure (Endress & Mehler, 2009).

While most neuroimaging studies of derivational morphology have examined linear structures (e.g., Meinzer et al., 2009; Pliatsikas, Johnstone, & Marinis, 2014), there is also evidence from non-linearly derived Hebrew words (Bick et al., 2008; Bick et al., 2010; Bick et al., 2011; Bitan et al., 2020) that processing the root morpheme involves left frontal areas, including dorsal LIFG (pars opercularis and pars triangularis) and left MFG. However, previous studies have not directly compared words with a linear and non-linear structure. Participants in the current study have experience with both linear and non-linear structures of derived words from their native language. Nevertheless, the easier and more salient morphemes in the linear structure may result in differences in the neural mechanisms involved in learning them.

### Individual Differences in Learning and Consolidation

In addition to the general effect of the learner’s native language, individual differences in prior linguistic abilities may affect the neural mechanisms involved in learning a new language. Numerous studies have shown the role of prior linguistic and cognitive abilities in predicting behavioural outcomes of second language learning (Dörnyei, 2014; Ganschow et al., 1998; Koda, 2009; Morgan-Short et al., 2014; Zafar & Meenakshi, 2012). More specifically, morphological awareness, which is the ability to consciously recognize and manipulate morphemes (Carlisle & Feldman, 1995; Ke et al., 2021) has been shown to play a critical role in L2 literacy (Ramirez et al., 2010; Wang et al., 2006) and vocabulary development (Pasquarella et al., 2011). A study of L2 English Grade 4 students showed a strong relation between derivational morphological awareness and vocabulary knowledge (Kieffer & Lesaux, 2012). Similarly, phonological awareness in L1, which involves the ability to recognize and manipulate the units of sound in a word, has been found to predict better word learning (Hu, 2014), reading comprehension (Melby-Lervåg et al., 2012), general language proficiency (Borodkin & Faust, 2014), and better consolidation of linguistic information (Ben Zion et al., 2019; Bitan & Booth, 2012; Landi et al., 2018) in a novel language. Neuroimaging studies also show that higher metalinguistic abilities in L1, such as phonological awareness and rapid naming, are related to neural activity involved in the processing of new words in an L2 (Cao et al., 2017). In line with these findings, the current study will examine the relation between L1 morphological and phonological abilities and the neural mechanisms involved in learning and generalizing morphological regularities from a novel morpholexicon.

### The Current Study

In this study we examined the role of decomposition in the early stages of learning morphologically derived words in an artificial morpholexicon. These words were learnt in an L2 setting: They were paired with familiar concepts and with their L1 translations. We used fMRI to scan Hebrew-speaking individuals before and after a multisession training program that involved learning a novel set of words that are morphologically decomposable and are interrelated via derivation. We compared two types of morphologically derived words: linear and non-linear structure, and also compared them to simple monomorphemic words (i.e., words that cannot be decomposed). Our behavioural results from tasks performed outside the scanner in the same study, which included more participants (Nathaniel et al., 2023), revealed successful decomposition with both linear and non-linear morphology. This was evident by better generalization to untrained words with a complex morphological structure in comparison to morphologically simple words, and better generalization of the linear compared to the non-linear complex words by the end of four training sessions. Learners also exhibited better overall performance for trained words with a linear structure in comparison to non-linear and simple words during training, suggesting that linear structure is easier to learn. We expect to see similar behavioural results in tasks performed during scanning in the present study.

For the fMRI results, we had four specific preregistered hypotheses (https://osf.io/ju7vh/): First, in line with previous studies implicating the dorsal LIFG (pars opercularis and pars triangularis) in morphological decomposition of derived words (Bitan et al., 2020; Leminen et al., 2019), we predicted that for trained words, both types of morphologically complex words, which can be decomposed, will reveal greater activation in dorsal LIFG in comparison to simple words. Second, because we hypothesized that the morphological structure of complex words would facilitate generalization to unfamiliar words, we predicted that, when processing untrained words during the last session, morphologically complex words would show greater activation in comparison to simple words in frontal regions associated with decomposition, as well as in temporal regions associated with lexical representations. Third, in line with the CLS model (Davis & Gaskell, 2009; McClelland et al., 1995), we predicted that learning and consolidation of the morphologically complex words, compared to simple words, would show a greater increase in reliance on cortical areas, accompanied by a decrease in reliance on hippocampal activation, from the first to the last session. Fourth, in line with previous language learning studies showing that better linguistic abilities, such as phonological awareness or vocabulary, predict better consolidation of linguistic information (Ben Zion et al., 2019; Bitan & Booth, 2012; Landi et al., 2018), we predicted that morphological and phonological abilities in L1 (Hebrew) would correlate with an increase in activation from the beginning to the end of training in frontal and temporal regions, specifically for the morphologically complex words.

## MATERIALS AND METHODS

The study hypotheses, criteria for data exclusion, and analysis plans were all preregistered through the Open Science Framework prior to examining the data or completing the data collection, and no deviations were made from the preregistration.

### Participants

Thirty-one native Hebrew speakers (13 males, age range: 21.84–32.06 years, *M* = 26.90, *SD* = 2.86) were recruited for the current study, and they were all included as part of a larger sample in a previous paper reporting the behavioural results of tasks performed outside the scanner (Nathaniel et al., 2023). The sample size was determined based on prior studies that examined morphological effects using fMRI, with comparable or smaller sample sizes (Bick et al., 2011; Bozic et al., 2013; Davis et al., 2009; Kimppa et al., 2019; Koester & Schiller, 2011; Nevat et al., 2017). Considering a potential dropout rate of approximately 25%, we aimed to achieve our preregistered target of 25 participants by initially recruiting 31 participants. All participants demonstrated proficiency in Hebrew and identified it as their primary language, as confirmed through the Language Experience and Proficiency Questionnaire (LEAP-Q; Marian et al., 2007; Prior, 2012). In addition to Hebrew, all participants, consistent with the broader population of young adults in Israel, also spoke English, which they had learned through formal classroom instruction. Furthermore, 16 of the participants disclosed exposure to one or more additional languages. Participants who had been exposed to English or any of the other languages within their home environment or through immersive experiences, such as residing abroad for more than 4 months during childhood or one year as an adult (as assessed based on the LEAP-Q), were excluded from the study. These criteria were assessed during the recruitment process, and one participant was subsequently excluded based on these specific conditions after their initial participation in the study. The average age participants started learning L2 (English) was 8.4 years, *SD* = 1.8. Participants also completed questions regarding their mean percentage of exposure to L1 and L2, and a subjective assessment of their language proficiency on a 10-point scale, averaged over speaking, listening comprehension, and reading. All participants were right-handed, with no history of neurological or psychiatric disorders, sleep disorders, or learning disability. Two more participants were excluded from the group analysis based on our preregistered criteria for data exclusion (described below); this resulted in 28 participants who were included in the final analysis. Table 1 shows the mean, standard deviation, and range for variables from the LEAP-Q and background tests for these 28 participants.

**Table 1.** Descriptive statistics for each background test (*n* = 28)

Test	Unit of measure	Mean (*SD*)	Range
*Language Experience and Proficiency Questionnaire*			
Exposure to L1	%	82.7 (13.0)	50.0–98.7
Exposure to L2	%	15.9 (12.6)	1.0–50.0
Proficiency in L1	10-point scale	9.7 (0.5)	8.0–10.0
Proficiency in L2	10-point scale	6.7 (2.2)	0.0–9.7

*Screening tests*			
Phoneme deletion time	Total time (min)	1.9 (0.4)	1.3–2.8
Non-word reading	Number of correct words in a minute	6.5 (1.2)	5.0–10.0
Morphological fluency	Mean of correct words from 10 targets	3.1 (1.1)	1.4–6.8
Morphological relations	Number of correct words (max score of 20)	16.5 (2.7)	11.0–20.0
Digit span (forward)	Number of correct sequences (total of 16 trials)	10.3 (2.1)	6.0–14.0

In order to ensure that participants fell within the typical range for reading ability, short-term memory (STM), and intelligence, we used the following screening assessments: Reading words per minute (Shatil, 1997), digit span (Wechsler, 1997), and Matrices (Wechsler, 1999). Given the absence of standardized reading tests tailored to adult Hebrew speakers, we utilized the aforementioned tests in conjunction with locally derived norms obtained from an independent sample of 191 individuals at the University of Haifa. This approach aligns with the methodology used in previous research studies (Ben Zion et al., 2019; Bitan et al., 2020; Weiss et al., 2015; Weiss et al., 2016). None of the 28 participants had a score lower than 1.5 standard deviations below the norm, which was our exclusionary criterion for the above screening tests.

Additional language tests were used to assess Hebrew phonological awareness. One was a phoneme deletion test (Ben-Dror & Shany, 1996), in which participants were instructed to listen to 25 pseudo-words and repeat them while omitting a specified phoneme at the beginning or middle of the given pseudo-word; total time (in min) to accurately read the words was computed. A non-word reading test (Shatil, 2002), in which participants read a list of non-words with diacritic marks as quickly and accurately as possible within a minute was also given; the number of correct non-words read within a minute was calculated. A phonological composite score was created by averaging standardized scores on the non-words per minute and phoneme deletion tasks per participant. This composite score has previously shown to correlate with early consolidation when learning morphological inflections (see Ben Zion et al., 2019).

Morphological awareness in Hebrew was assessed using tests that rely on the decomposition of Semitic roots and templates. In a test for morphological fluency (Leikin & Hagit, 2006), participants heard 10 target words, and for each target word they were asked to say as many words as they could that share the same vocalic template within 30 seconds. For example, for the target word *mélex* (king), the template used is CéCeC (C = consonant). Participants were required to generate words based on the given vocalic template (regardless of whether these words have a productive root), such as *kélev* (dog), *kéter* (crown), *séfer* (book), *Šemeš* (sun), and so forth. The total count of correctly produced words, averaged across the 10 target words, was calculated. In the morphological relations test (Leikin & Hagit, 2006), participants heard 20 pairs of words and had to determine whether these pairs shared the same tri-consonantal root. It is important to note that all word pairs were chosen to be semantically unrelated. For example, the words *taklit* (record) and *miklat* (shelter), which do not share the same semantic associations and connotations, share the root morpheme k-l-t (take in), and the answer is “yes,” while the words *méšek* (farm) and *mašké* (a drink) are phonologically similar but in fact have different roots (m-š-k and š-k-h; in *méšek* the initial consonant “m” is part of the morphological root while in *mašké* it is part of the template), and the correct answer is “no.” The number of correct responses were counted, with a maximum score of 20. While these tests cover only some aspects of derivational morphology in Hebrew, Leikin and Hagit (2006) report that these morphological tests are highly correlated with each other (with weights of 0.848 and 0.887 in a principal component analysis). In addition, the morphological relations task has previously shown to be correlated with brain activation during the reading of morphologically complex and simple words in Hebrew (Bitan et al., 2020). Composite scores from both phonological and morphological tests were used to compute correlations with activation changes.

Lastly, STM was assessed using the digit span forward task (WAIS II Heb; Wechsler, 1997), where participants were asked to recall a sequence of orally presented digits of increasing length (two trials per sequence length), with a total of 16 trials. Testing was discontinued if participants failed to repeat two trials of the same length. Total score was calculated as the number of sequences that were recalled in the correct order.

### Procedures

The study received approval from the Helsinki committee at Sheba Medical Center, and all participants provided their written informed consent prior to their participation. Participants underwent five experimental sessions. In the initial screening session, participants filled out demographic surveys, as well as a series of screening and language tests. The subsequent four sessions (referred to as S1–S4) occurred within a week. Specifically, S1 and S2 were consistently 48 hours apart, while the time gap between the other sessions ranged from 48 to 72 hours, in each session participants were trained and tested on novel words in an artificial language. During S1 and S4, which lasted approximately 2.5 hours, participants completed behavioural training followed by additional blocks of testing in an MRI scanner at the Strauss Computational NeuroImaging Center at Tel Aviv University. During S2 and S3, which lasted approximately an hour, participants completed behavioural training and testing only at the University of Haifa.

### Stimuli

During the training sessions, participants were trained in an artificial morpho-lexicon (named Boodronibi) developed for the purpose of this study. Participants learned nouns from three morphological conditions (see Table 2).

1. **complex non-linear (CNL)**: This morphologically complex condition has non-linear structure. Words in this condition were composed of a set of novel four-consonantal roots that carried basic concrete meanings (e.g., G-L-B-K = “fish,” K-V-M-Š = “book”; for more examples see Table 2), with four different vowel templates that do not exist in Hebrew and that were designed to provide more abstract semantic category information (*CuCCáCi* = person, *CoCCéCu* = place, *CéCCiC* = large number, *CíCCoC* = thing). The interleaving of these roots and templates together created word forms that were derivationally related based on their internal structure (e.g., *kuvmáši* = “novelist”; *kovméšu* = “library”; *kévmiš* = “book collection”; *kívmoš* = “bookmark”).
2. **complex linear (CL)**: This morphologically complex condition has linear structure. Words in this condition consisted of a set of novel monosyllabic roots with concrete meanings (e.g., *zomg*- = “bird,” *kest*- = “sheep”) and a set of suffixes that do not exist in Hebrew (or English), each indicating the same abstract semantic categories listed above (e.g., *kestíne* = “shepherd,” *kestápa* = “sheep pen,” *késtom* = “flock of sheep,” *késtuk* = “wool”). The concatenation of these roots and suffixes created derivationally related word forms based on their linear structure.
3. **simple (S)**: In this morphologically simple condition, words have a semantic structure that is similar to that of the words in the two complex conditions, but they have no internal morphological structure (e.g., *zéfmok* = “insect exterminator,” *šumbúsa* = “insect zoo,” *dínzet* = “swarm of insects,” *páldun* = “insect repellent”). This condition served as a baseline for comparison with the complex conditions.

**Table 2.**
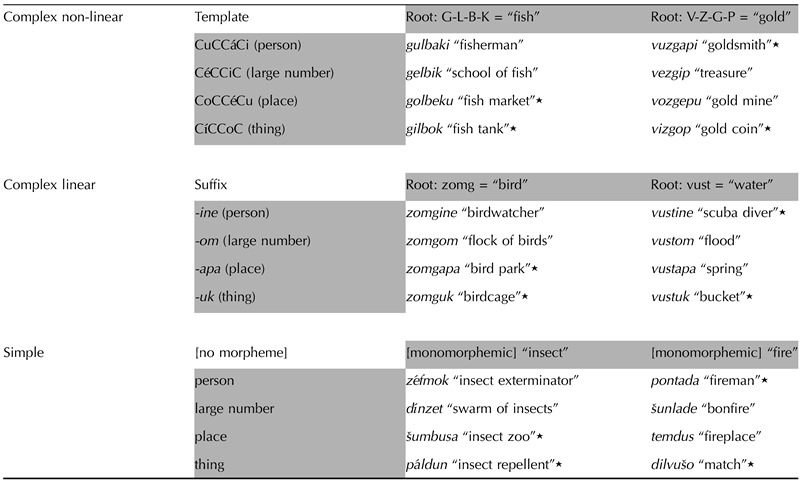
Sample words from the three morphological conditions for trained and untrained words

*Note*. Untrained words are marked with a star. The stimuli included 72 trained words and 36 untrained words.

While all the conditions had similar semantic relations among words, only for the morphologically complex conditions were there also morphological relations between different words, based on either the root or the template/suffix, as illustrated in Table 2.

The linguistic structure of both types of complex words, in which the root of a word represents a basic semantic concept and the suffix/template represents the semantic category, can be observed across a wide array of natural languages (e.g., English, Hebrew, German, Arabic). The use of a variety of morphological strategies to show semantic contrasts is also characteristic of many natural languages. For example, English relies on all three strategies when building families of semantically related words—linear (e.g., ample–amplify), non-linear (e.g., food–feed), and using morphologically unrelated words (e.g., thief–steal)—albeit with different frequencies than Boodronibi.

A total of 36 Boodronibi words were created for each experimental condition. Within each condition, 24 of these words were presented during the training phase, containing a mix of 12 root morphemes combined with four template/suffix morphemes. In the final session, a generalization test included the remaining 12 words per condition. These additional words were constructed by combining familiar morphemes from trained words in novel combinations (see Table 2). Word meanings were designed to be consistent across the conditions as follows: The root meanings in the three conditions were organized in triplets, ensuring that the meanings of all roots within a triplet belonged to the same semantic category, for example, fish-bird-insect ➔ animals; gold-water-fire ➔ elements (in traditional folk understanding); and so on. The meanings within the triplets were counterbalanced between conditions among participants. The template/suffix represented one of four abstract semantic categories, namely, person, large number, place, and thing. These meanings remained consistent between conditions (e.g., the template *CuCCáCi* and the suffix -*ine* both represented person). The simple condition included these semantic categories, but the words lacked internal morphological structure. Across all conditions, the words were comparable in terms of length, internal syllable structure, and number of syllables, with half of the words in each condition having a CVCCVC structure, and the other half having a CVCCVCV structure. This ensured equivalent learning conditions.

Auditory presentation of Boodronibi words was carried out using a recorded female voice. In order to enable comparisons of performance of Hebrew speakers with American English speakers in a future study, we created a mixed accent that would be perceptible but dissimilar from their respective native accents, with an American-English-like place of articulation for the consonants, Hebrew-like quality for the vowels, and avoidance of vowel reduction. The recorded stimuli underwent processing using PRAAT (Boersma & Weenick, 2018) for trimming recordings and adjusting intensity to 76 dB. Duration adjustments were made using WOSLA (a program written by Yizhar Lavner from Tel-Hai College), resulting in an average duration of 937 ms (Range: 740–1,126 ms). Pairwise *t* tests were conducted to confirm that there were no significant duration differences between conditions (i.e., CNL vs. CL, CNL vs. S, and CL vs. S), considering syllable length for both trained and untrained words separately (Trained–Duration: *p*s > 0.416; Untrained–Duration: *p*s > 0.309). Native Hebrew speakers transcribed the recordings to ensure clarity of pronunciation.

Each Boodronibi word was accompanied by a Hebrew translation and a Hebrew definition. The translation consisted of a 1-2-word Hebrew term describing the word, while the definition included a brief sentence explaining the Hebrew meaning of the word. For instance, the word *gulbaki* was translated as “fisherman” and defined as “somebody who fishes.” The word *zomgine* was translated as “birdwatcher” and defined as “somebody who spots birds.” In all pairwise comparisons between conditions (i.e., CNL vs. CL, CNL vs. S, and CL vs. S), translations for trained and untrained words were matched in terms of the total number of words, total number of syllables, total number of morphemes, and average word frequency (Hebrew Corpus Web 2014, heTenTen14; Sketch Engine, 2014). The order of the conditions and the pairing of auditory items and translations were counterbalanced among participants.

### Behavioural Tasks

Participants completed four sessions of training on the Boodronibi words (S1–S4), which included the following phases:

1. **familiarization**: In S1, participants heard each Boodronibi word and saw its translation and definition written on the screen in Hebrew (5,000 ms). Participants were told they would learn words in a novel lexicon, and were asked to repeat the Boodronibi word aloud. Vocal responses were recorded in all phases. Across all phases, Boodronibi words were never presented visually.
2. **training**: In S1–S4, participants heard each Boodronibi word and were asked to repeat the word aloud. Four written Hebrew translations then appeared on the screen: a correct translation and three distractors that were translations of other trained words. Two of these distractors were translations of words that were morphologically unrelated to the target word, while the third distractor was related to the target either by the meaning of the root (half of the trials), or by the meaning of the template/suffix (other half of the trials), and a corresponding manipulation was maintained in the simple condition. Participants then chose the correct translation by pressing one of four keys (which corresponded to the location of the translations displayed on the screen). Following their responses, participants received visual feedback showing the correct translation (1,500 ms). Participants were trained across three blocks per session, with each word appearing once per block.
3. **test**: In S1–S4, participants heard each Boodronibi word once and were asked to repeat it aloud. Four written Hebrew translations appeared on the screen, and participants chose the correct response by pressing a key on the keyboard. Feedback was not provided during testing. Participants’ accuracy served as a measure of their explicit knowledge of the meaning of trained words. Each word only appeared once, i.e., 24 items per condition in each test. Each session included two tests, T1 (before training) and T2 (after training).
4. **generalization**: In S4, after completing T2 of trained words, participants were presented with a familiarization block (similar to S1), for the 36 untrained items. These words were then tested in a generalization test, which was similar to the test of trained items.

### fMRI Tasks

At the end of S1 and S4, participants performed two tasks in the fMRI scanner: an auditory repetition task and a translation recognition task.

#### Auditory repetition task

Participants were asked to orally repeat each word as fast and accurately as possible. Results from this task will be presented elsewhere.

#### Translation recognition task

On experimental trials, participants heard each word and saw a black fixation, followed by a translation. Half of the trials presented a correct translation and half presented an incorrect translation. Half of the incorrect translations had a shared root and the other half a shared template/suffix (and a corresponding manipulation was maintained in the simple condition). Participants were asked to judge the correctness of the translation by either pressing “1” using their index finger to denote the translation was correct, or pressing “2” using their middle finger if the translation was incorrect. Responses were recorded from the end of the word presentation. On perceptual control trials, participants heard static noise and saw a black fixation, and then saw “continue,” and were instructed to press “1” using their index finger. On the baseline trials, participants saw two black asterisks, and were instructed to remain still while they waited for the next trial. Seventy-two experimental trials were intermixed with 24 perceptual and 24 baseline trials, and jittered with 1.6% time of null. A total of 720 trials (480 trials for trained and 240 trials for untrained) were acquired across six runs of 6:58 mins, that is, two runs each in S1 and S4 for trained words, and two runs in S4 for untrained word. All items were balanced to be presented with correct and incorrect translations in each run.

### fMRI Data Acquisition

Images were acquired using a 3T Siemens Magnetom Prisma scanner. Visual stimuli were projected onto a screen and viewed through a mirror attached to the head coil, and auditory stimuli were presented through in-ear headphones. Participants’ responses were recorded using an optical microphone (FOMRI-II, Optoacoustics, Mazor, Israel) and a response box. Functional images were acquired using the echo planar imaging method. For each volume, 56 slices that cover the whole brain were collected using a multiband (acceleration factor = 2) interleaving sequence (voxel size = 2.25 × 2.25 × 2.25 mm thickness, repetition time [TR] = 2,000 ms, echo time [TE] = 30 ms, field of view [FoV] = 216 mm, flip angle = 72°). Anatomical images were collected via high resolution T1-weighted images (voxel size = 1 × 1 × 1 mm, TR = 1,750 ms, TE = 2.45 ms, FoV = 224 mm, flip angle = 8°). Each sequence began with 8 seconds of dummy and calibration scans (4 volumes) to allow for the MRI signal to stabilize and concluded with an additional 6 seconds (3 volumes) to allow for the measurement of the blood oxygen level dependent response associated with the last trial.

### fMRI Preprocessing

fMRI data were preprocessed using the Statistical Parametric Mapping toolbox for Matlab (SPM12 – Welcome Trust Centre for Neuroimaging, University College London, www.fil.ion.ucl.ac.uk/spm). The first four volumes collected in each functional run were discarded. Images were realigned to the mean functional image across runs. Anatomical images were segmented and then combined with the participant’s raw anatomical (with segmented grey matter, white matter, and cerebral spinal fluid), to create a skull-stripped image. Functional images were then registered to the skull-striped anatomical image. Using the deformation field generated from segmentation, functional images were normalized to the MNI (Montreal Neurological Institute) space, and then smoothed using a 6-mm isotropic Gaussian kernel. Lastly, ArtRepair toolbox (Mazaika et al., 2009) was used to identify outlier volumes among the functional images (using the “art_motionregress” and “art_global” parameters).

### Criteria for Data Exclusion

The following preregistered criteria were used to select the final sample for the current study:

- Participants who did not make a response on at least 15% of trials on the behavioural tasks were discarded (n excluded = 1).
- Across all training sessions, participants performing below 2 standard deviations in total learning from pre to post test (S4T2–S1T1) were excluded from the analysis (n excluded = 1).
- ArtRepair was implemented to correct for participant movement, as well as to detect and repair outlier volumes. These were defined as those with head movements exceeding 4 mm in any direction or deviations of more than 1.5% from the mean global signal. Based on these parameters, we excluded runs with more than 10% of repaired volumes (n excluded = 0).
- If there were problems with the scanner or the presentation software crashed, then the affected runs were repeated if there was available scanner time. Participants who failed to complete at least two of the four runs in the translation recognition task (for trained items) were replaced. Participants who did not respond on 30% of the language trials in any run were to be excluded from the analysis (n excluded = 0).

### Whole-Brain Analysis

Preprocessed data were analyzed at the first level using a general linear model (GLM) for event-related designs. Only correct responses were included in the analysis. The model included regressors for the three morphological conditions (complex non-linear, complex linear, and simple words), perceptual control, fixation baseline, and errors (incorrect responses). Contrast maps were generated for each experimental condition versus perceptual control, as well as for comparisons between the conditions (CNL > NL; CNL > S; CL > S) across sessions, as well as in the first (S1) and last (S4) training sessions separately. At the second level, a one-sample t test was used for group level analysis to examine activation in each condition against perceptual control, as well as for comparisons between the morphological conditions. In addition, paired t tests were used to examine the effects of session (S1 vs. S4) in each morphological condition. Statistical maps are depicted at familywise error (FWE) corrected threshold of *p* < 0.05, and cluster extent threshold of k ≥ 10 voxels.

#### Region-of-interest analysis

Region of interest (ROI) analyses were conducted at the second level using six basic contrasts from the first level, comparing each of the three morphological conditions to perceptual control, separately for S1 and S4. Four ROIs were defined in the left hemisphere in regions that have been shown to be involved in morphological processing, word learning, and consolidation. (1) Hippocampus (Bein et al., 2019; Breitenstein et al., 2005) was defined via an anatomical mask based on the Automated Anatomical Labeling (AAL) atlas (Tzourio-Mazoyer et al., 2002), split in approximately three equal lengths along the *y*-axis. (We looked at the anterior and posterior thirds: the posterior portion of the hippocampus from Y = −40 to −30 and anterior hippocampus from Y = −18 to −4; Collin et al., 2015.) (2) LIFG (Bozic et al., 2007; Leminen et al., 2016; Nevat et al., 2017) was defined anatomically based on the AAL atlas and included all three subregions: pars opercularis, pars triangularis, and pars orbitalis. (3) Left STG (Bozic et al., 2013) was defined as an anatomical mask based on the AAL atlas, and split in two approximately equal lengths along the *y*-axis, that is, posterior portion of the STG from Y = −54 to −24 and anterior portion of the STG from Y = −22 to 6. (4) Left MTG (Bozic et al., 2013) was defined as an anatomical mask based on the AAL atlas and was split in approximately equal lengths along the *y*-axis, that is, posterior portion of the MTG from Y = −76 to −34 and anterior portion of MTG from Y = −32 to 10.

The top 100 most active voxels for each morphological condition > perceptual control (**** - say “pass”) within the ROI masks were selected based on *t* values of that contrast, separately for each individual. Beta values associated with each condition were then extracted using the MarsBaR toolbox for SPM (Brett et al., 2002). This enabled us to select voxels that were most responsive and sensitive to the experimental manipulation and were therefore more accurate in detecting neural effects. Statistical analyses were carried out using IBM SPSS Statistics Software (Version 25). Separate repeated-measures GLM analyses were conducted for runs of trained and untrained items, for each ROI. In all analyses, average beta values extracted from the top 100 active voxels for each morphological condition (CNL, CL, S) versus perceptual control were the dependent variable, with condition (CNL, CL, and S), session (S1 vs. S4—only for trained items), and subregions (anterior and posterior subregions in STG, MTG, and hippocampus, and 3 IFG subregions) as the within-subject factors.

Based on our preregistered analysis plans, we also conducted correlational analyses to examine whether activation extracted in each ROI and in each condition correlated with: (1) behavioural performance in the experimental task and (2) prior linguistic abilities. As we had different hypotheses for each ROI for the correlations (LIFG, STG, MTG, hippocampus), we applied a Bonferroni correction to account for multiple comparisons across the three morphological conditions and for the number of subregions within each ROI. This was done as follows: IFG (3 subregions × 3 conditions), *p* = 0.05/9 = 0.006; STG (2 subregions × 3 conditions), *p* = 0.05/6 = 0.008; MTG (2 subregions × 3 conditions), *p* = 0.05/6 = 0.008; and hippocampus (2 subregions × 3 conditions), *p* = 0.05/6 = 0.008.

##### Behavioural performance in experimental tasks.

We used SPSS to calculate Pearson correlations between brain activation in each morphological condition (morphological condition > perceptual control) with performance on the behavioural tests conducted outside the scanner before and after training on the 4-alternative forced choice (4-AFC) translation selection task, as they afforded correlations with more nuanced behavioural measures. We correlated the difference in brain activation between the first and last sessions (S4–S1) with

- total improvement during training, i.e., total learning, measured as the difference between the first and last behavioural tests (S4T2–S1T1)
- offline gains after the first training session, i.e., consolidation, measured as the difference between the last test in Session 1 and first test in Session 2 (S2T1–S1T2)
- performance on untrained items on the last session (S4 UT)

##### Prior linguistic abilities.

We also examined if there was an effect of participants’ L1 linguistic abilities on neural changes associated with the learning of the new words. We correlated differences in brain activation between the first and last scans (S4–S1) with

- phonological abilities measured using a phonological composite score, which was calculated by computing standardized scores for each participant on the non-word reading and phoneme deletion tasks and then averaging the two standardized scores per participant
- morphological abilities measured using a morphological composite score, which was calculated by similarly combining standardized scores on the morphological fluency and morphological relations tests for each participant

#### Sensitivity analysis

Lastly, given our sample size, we conducted post hoc sensitivity analysis in G*power 3.1 (Faul et al., 2007), to determine the minimum reliable effect size for each of our analyses, using an alpha of 0.05 and a power of 80%.

## RESULTS

Results are presented in four sections, we first report behavioural results (accuracy and response time) from the translation selection task performed outside the scanner and the translation recognition task performed inside the scanner, followed by the preregistered fMRI analyses, the exploratory analyses, and the sensitivity analysis.

### Behavioural Results

#### Trained items outside the scanner (4-AFC translation selection task)

**Figure 1.**
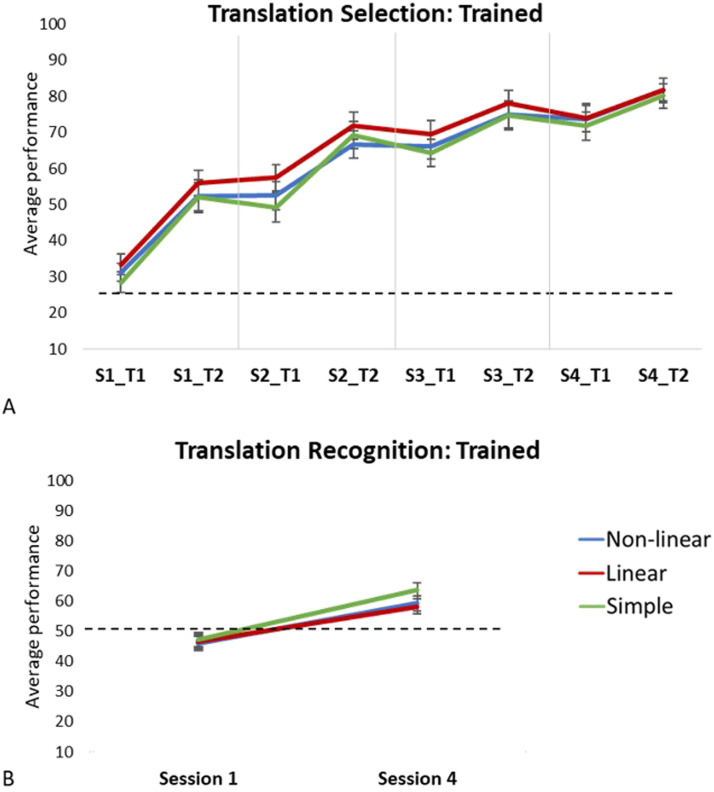
Mean percentage accuracy for trained items performed (A) outside the scanner (translation selection task) and (B) inside the scanner (translation recognition task), separately for each morphological condition. Black dotted line indicates chance-level performance. S = session, T = test.

Here we report results for the 28 participants included in the analysis of the fMRI task (these are a subset from a larger sample of participants that performed the behavioural tasks, described in Nathaniel et al., 2023). One-sample *t* tests showed that participants performed above chance level (25%) on the first test (S1–T1) for both complex conditions but not the simple condition (complex non-linear: *t* = 2.58, *p* = 0.016; linear: *t* = 2.90, *p* = 0.007; simple: *t* = 1.20, *p* = 0.239). However, performance was significantly above chance for all three conditions on the second test in the first session (S1–T2: all *t* ≥ 6.06, all *p* < 0.001), see Figure 1A. We ran a repeated-measures analysis of variance (ANOVA) on the participants’ performance on the eight tests performed before and after each training session, with accuracy as a dependent measure and the following within-subject factors: morphological condition (complex non-linear, complex linear, simple), session (4 testing sessions, S1–S4), and test (T1, pre-training vs. T2, post-training). The analysis showed a significant main effect of session, *F*(3, 78) = 244.08, *p* < 0.001, *η*_*p*_^2^ = 0.904, indicating an increase in accuracy across sessions, as well as a main effect of test, *F*(1, 26) = 242.33, *p* < 0.001, *η*_*p*_^2^ = 0.903, suggesting an increase in accuracy between T1 and T2 within sessions. There was also a significant main effect of morphological condition, *F*(2, 52) = 4.73, *p* = 0.013, *η*_*p*_^2^ = 0.154. Pairwise comparisons with Bonferroni correction revealed that accuracy on the complex linear condition was higher than the simple condition (*p* = 0.025). There was no significant difference between the other conditions. We also found a significant interaction between session and test, *F*(3, 78) = 12.89, *p* < 0.001, showing that the improvement in performance between T1 and T2 within each session, decreased between subsequent sessions, S1–S2: *t*(27) = 2.27, *p* = 0.032; S2–S3: *t*(27) = 4.30, *p* < 0.001; S3–S4: *t*(27) = 0.542, *p* = 0.592.

Because participants’ speed of response can reflect the ease at which they access representations of their acquired knowledge, and to examine if there were any speed–accuracy trade-offs, we also ran a repeated-measures ANOVA with reaction time (RT) as a dependent measure. Analysis revealed main effects of session, *F*(3, 81) = 28.155, *p* < 0.001, *η*_*p*_^2^ = 0.510, and test, *F*(1, 27) = 58.89, *p* < 0.001, *η*_*p*_^2^ = 0.686, suggesting a significant decrease in RT across sessions, as well as decrease in RT between T1 and T2 within sessions, respectively. There was no main effect of condition. A three-way interaction between session, test, and condition was significant, *F*(6,162) = 4.107, *p* = 0.001, *η*_*p*_^2^ = 0.132. Follow-up analyses using repeated-measures ANOVAs were conducted for each condition separately. Only the simple condition showed a significant interaction of session and test, *F*(3, 81) = 4.28, *p* = 0.007, *η*_*p*_^2^ = 0.137, revealing a larger within-session decrease in RT in the second compared to the first session, S1–S2, *F*(1, 27) = 7.68, *p* = 0.010, *η*_*p*_^2^ = 0.221. There was no difference in within-session improvement between other sessions, S2–S3, *F*(1, 27) = 2.55, *p* = 0.122, and S3–S4, *F*(1, 27) = 0.021, *p* = 0.885. There was also no significant Session × Test interaction for the other conditions, non-linear, *F*(3, 81) = 0.481, *p* = 0.697, and linear, *F*(3, 81) = 1.88, *p* = 0.139.

#### Trained items inside the scanner (translation recognition task)

Participants’ performance in the first scan did not exceed chance level (50%) for any of the conditions, but performance was significantly above chance in Session 4 for all conditions (all *t* ≥ 3.54, all *p* ≤ 0.001; see Figure 1B). We conducted a repeated-measures ANOVA for the translation recognition task with accuracy as the dependent variable and the following within subject factors: session (S1 vs. S4) and morphological condition (complex non-linear, complex linear, simple). The analysis revealed a main effect of session, *F*(1, 27) = 85.39, *p* < 0.001, *η*_*p*_^2^ = 0.760, suggesting an increase in accuracy from the first to the last session. There was also a significant main effect of condition, *F*(1, 27) = 3.25, *p* = 0.042, *η*_*p*_^2^ = 0.218. While visual inspection of Figure 1B suggests higher accuracy on the simple condition compared to the complex conditions, pairwise comparisons with Bonferroni correction revealed no significant difference between any of the conditions.

In order to test whether participants’ performance on the translation recognition task in the scanner reflected their knowledge measured with the translation selection task outside the scanner, we also tested the correlation between accuracy on both tasks, averaged across sessions, separately for each condition. Performance outside the scanner highly correlated with performance within the scanner, in each morphological condition, non-linear, *r* = 0.579, *p* = 0.001; linear, *r* = 0.608, *p* = 0.001; and simple, *r* = 0.747, *p* < 0.001. Additional analysis and discussion regarding the discrepancy between performance on the recognition task performed inside the scanner and the translation selection task performed outside the scanner is provided in the Supporting Information, available at https://doi.org/10.1162/nol_a_00150 (see Figure S1).

An analysis with RT as the dependent variable showed a main effect of session, *F*(1, 27) = 29.63, *p* < 0.001, *η*_*p*_^2^ = 0.523, suggesting a decrease in RT from the first to the last session. There was no main effect of condition.

#### Untrained items outside the scanner (4-AFC translation selection task)

**Figure 2.**
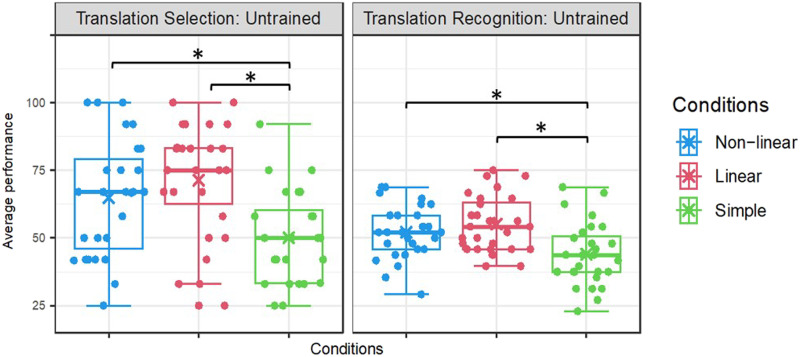
Mean percentage accuracy for untrained items performed outside the scanner (left panel: translation selection task) and inside the scanner (right panel: translation recognition task), separately for each morphological condition. Asterisks denote significant effects in paired comparisons of the morphological conditions (*p* < 0.05). The “X” within the boxplot denotes mean performance across subjects, centre line denotes median value (50th percentile), box contains 25th to 75th percentiles, and whiskers mark the 5th and 95th percentiles.

A repeated-measures ANOVA for performance on untrained items with accuracy as the dependent measure and morphological condition as the within-subject factor, revealed a significant main effect of condition, *F*(2, 54) = 21.62, *p* < 0.001, *η*_*p*_^2^ = 0.445. Pairwise comparisons with Bonferroni correction revealed significantly higher accuracy on both complex conditions compared to the simple condition (see Figure 2, left panel): non-linear compared to simple (*p* = 0.001) and linear compared to simple (*p* < 0.001). There was no difference in generalization between the linear and non-linear conditions (*p* = 0.233).

Analysis with RT as the dependent variable showed a significant main effect of condition, *F*(2, 54) = 11.38, *p* < 0.001, *η*_*p*_^2^ = 0.296. Pairwise comparisons with Bonferroni correction revealed significantly faster RTs for the complex non-linear compared to simple condition (*p* = 0.002), as well as complex linear compared to simple condition (*p* = 0.002). There was no difference between the linear and non-linear conditions.

#### Untrained items inside the scanner (translation recognition task)

A repeated-measures ANOVA, with accuracy as the dependent variable and morphological condition as the within-subject factor, revealed a main effect of condition, *F*(2, 54) = 16.29, *p* < 0.001, *η*_*p*_^2^ = 0.376. Pairwise comparisons with Bonferroni correction showed that generalization in both morphologically complex conditions was significantly more accurate than in the simple condition: non-linear compared to simple, *p* = 0.003, and linear compared to simple, *p* < 0.001 (see Figure 2, right panel), and no difference between the linear and non-linear conditions.

Additionally, performance on the untrained words in the translation selection task correlated with performance on the untrained words in the translation recognition task within the scanner, for complex linear, *r* = 0.603, *p* = 0.001, and simple conditions: *r* = 0.441, *p* = 0.019, and marginally for the complex non-linear condition, *r* = 0.369, *p* = 0.053.

Analysis with RT as the dependent variable revealed no main effect of condition, *F*(2, 54) = 0.483, *p* = 0.620.

### Preregistered Analyses

#### Whole-brain analysis

**Figure 3.**
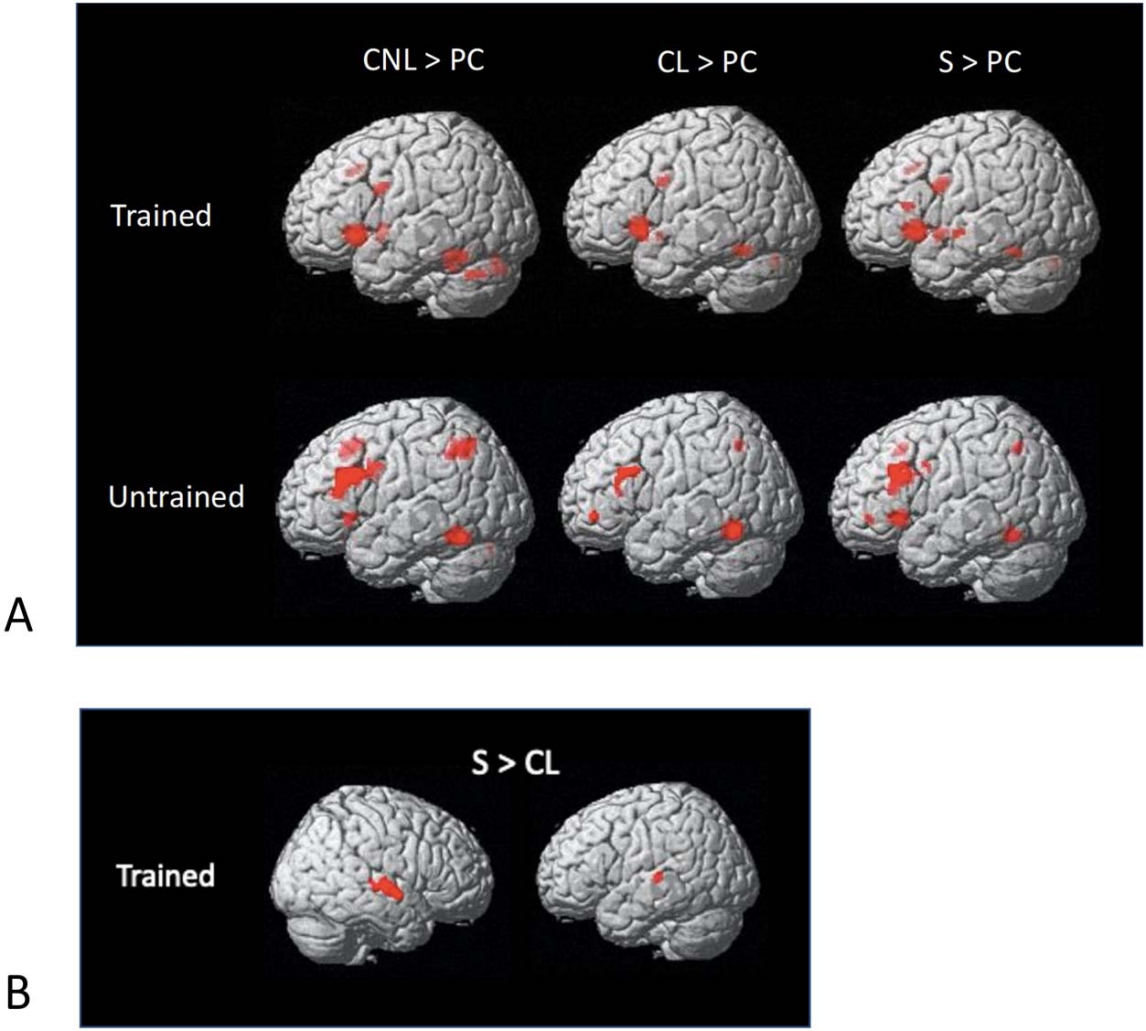
Activation maps. (A) Morphological conditions: complex non-linear (CNL), complex linear (CL), and simple (S) > perceptual control, for trained and untrained items on the translation recognition task. (B) Greater activation in the simple > complex linear condition, for trained items on the translation recognition task. Familywise error corrected *p* < 0.05, with cluster-size *k* ≥ 10.

Figure 3 and Table 3 show significant clusters of activation for each morphological condition (vs. perceptual control) for trained (across both sessions) and untrained words at the whole-brain level. We report significant effects after correcting for multiple comparisons (*p* < 0.05, FWE corrected, cluster extent threshold of k ≥ 10). The activation maps for all three conditions are very similar, showing fronto-temporal activation for trained items, and fronto-temporal and parietal activations for untrained items. We also assessed differences between morphological conditions across sessions and found one cluster showing a significant difference for the trained words between the Simple > Linear condition across sessions in bilateral STG (see Figure 3B). There were no differences between conditions for the untrained words, and no differences between sessions 1 and 4 in the trained words for any of the conditions.

**Table 3.** Regions showing activation in the whole-brain analysis for trained and untrained morphological conditions

Regions	BA	*Z* score	voxels	*x*	*y*	*z*
Trained items						
*CNL > PC*						
L supplementary motor cortex	6	6.52	191	−4	22	42
L inferior frontal gyrus: orbitalis	47	6.46	213	−34	18	−4
R insula	13	6.13	112	32	22	−2
L precentral gyrus	9	5.95	82	−44	4	34
L putamen	–	5.67	24	−26	2	2
L fusiform gyrus	37	5.64	55	−44	−50	−18

*CL > PC*						
L inferior frontal gyrus: orbitalis	47	6.29	215	−32	18	0
R anterior insula	13	6.26	113	32	22	−2
L superior frontal gyrus	12	5.71	88	−2	26	42
L precentral gyrus	9	5.45	47	−40	2	34
L inferior temporal gyrus	37	5.38	21	−46	−52	−18

*S > PC*						
R anterior insula	13	6.47	128	34	24	−4
L inferior frontal gyrus: triangularis	45	6.38	181	−6	18	48
L putamen	–	5.94	27	−28	4	−6
L precentral gyrus	9	5.63	59	−38	2	32

*Between condition comparison*						
*S > CL*						
L superior temporal gyrus	22	5.57	25	−54	−26	6
R superior temporal gyrus	22	5.55	69	66	−20	0

Untrained items						
*CNL > PC*						
L inferior frontal gyrus: triangularis	45	6.65	446	−42	30	14
L supplementary motor area	6	6.34	202	−2	22	44
L superior parietal lobule	5	5.99	144	−30	−62	46
L inferior temporal gyrus	37	5.74	98	−48	−54	−18
L anterior insula	13	5.73	46	−39	22	−4

*CL > PC*						
L inferior temporal gyrus	37	5.82	113	−48	−56	−18
L inferior frontal gyrus: opercularis	44	5.58	133	−50	18	26
L inferior frontal gyrus: orbitalis	47	5.2	14	−44	46	−4

*S > PC*						
L inferior frontal gyrus triangularis	45	6.19	187	−44	30	16
L inferior frontal gyrus orbitalis	47	5.85	128	−32	20	−4
L supplementary motor area	6	5.46	54	−2	22	44
R anterior insula	13	5.36	21	32	22	−6
L inferior temporal gyrus	37	5.18	20	−48	−56	−16
L precentral gyrus	9	5.16	18	−44	4	34
L superior parietal lobule	5	5.12	23	−30	−69	46

*Note*. Morphological conditions (complex non-linear (CNL), complex linear (CL), and simple (S)) > perceptual control (PC), and simple > complex linear condition. Familywise error corrected *p* < 0.05, *k* ≥ 10.

#### ROI analysis

We conducted separate ANOVAs for trained and untrained items on the translation recognition task, and for each of our ROIs, with average beta values extracted from the top 100 activated voxels for each morphological condition (CNL, CL, S) vs. perceptual control as the dependent variable. The within-subject factors were: condition (CNL, CL, and S), session (S1 vs. S4—only for trained items), and subregion (anterior and posterior subregions in STG, MTG, or hippocampus, or three subregions in IFG). All significant main effects and interactions are summarised in Table 4. Results from follow-up analyses on the interactions are presented by region in the following sections.

**Table 4.** Significant main effects and interactions in each region of interest for trained and untrained items on the translation recognition task

Main effect	*F*, *p*, *η*_*p*_^2^	Simple effect
Trained items		
*Left inferior frontal gyrus*		
Subregion	30.04, <0.001, 0.527	Triangularis > opercularis, triangularis > orbitalis

*Left superior temporal gyrus*		
Subregion	17.22, <0.001, 0.389	Anterior > posterior
Condition	10.78, <0.001, 0.285	CNL > CL, S > CL

*Left middle temporal gyrus*		
Condition	7.62, 0.001, 0.220	CNL > CL, CNL > S
Condition × Subregion	5.81, 0.005, 0.177	
Condition × Subregion × Session	3.09, 0.054, 0.103	

*Left hippocampus*		
Subregion	11.34, 0.002, 0.296	Posterior > anterior

Untrained items		
*Left inferior frontal gyrus*		
Subregion	43.65, <0.001, 0.618	Triangularis > opercularis > orbitalis
Condition × Subregion	2.54, 0.044, 0.086	

*Left superior temporal gyrus*		
Condition	6.64, 0.003, 0.197	S > CL

*Left middle temporal gyrus*		
Subregion	8.01, 0.009, 0.229	Posterior > anterior
Condition × Subregion	7.54, 0.001, 0.218	

*Note*. CNL = complex non-linear, CL = complex linear, S = simple.

##### Inferior frontal gyrus.

**Figure 4.**
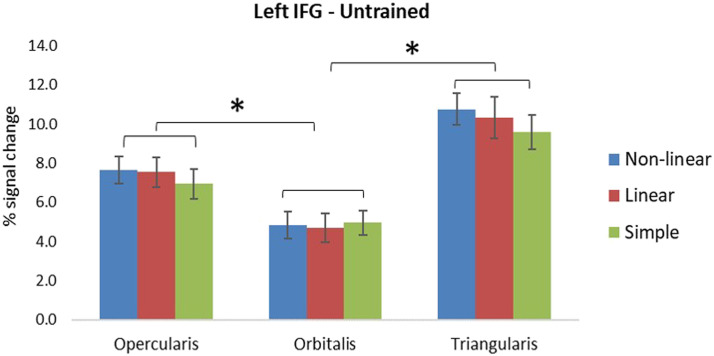
Percent signal change in the three subregions of the left inferior frontal gyrus (IFG) for untrained items on the translation recognition task for the three morphological conditions. Error bars indicate standard errors. Asterisks denote significant effects (*p* < 0.05).

For trained items, we found a significant main effect of subregion (see Table 4), with pairwise comparisons with Bonferroni correction revealing that activation in the triangularis was greater in comparison to the other two subregions of IFG, that is, opercularis (*p* < 0.001), and orbitalis (*p* < 0.001). For untrained items, we found a significant main effect of subregion (see Table 4), pairwise comparisons with Bonferroni correction revealed greater activation in triangularis compared to the other two subregions, that is, opercularis (*p* < 0.001), and orbitalis (*p* < 0.001), as well as greater activation in opercularis compared to orbitalis (*p* = 0.001). There was a significant interaction of condition and subregion (see Table 4), which revealed that the difference in activation between the complex non-linear and the simple condition was greater for opercularis, *F*(1, 27) = 5.45, *p* = 0.027, *η*_*p*_^2^ = 0.168, and triangularis, *F*(1, 27) = 6.37, *p* = 0.018, *η*_*p*_^2^ = 0.191, compared to orbitalis (see Figure 4).

##### Superior temporal gyrus.

**Figure 5.**
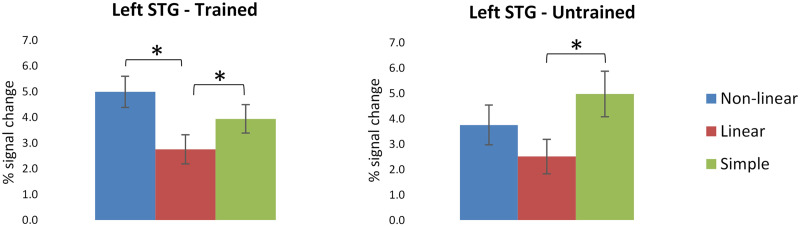
Percent signal change in the left superior temporal gyrus (STG) for trained and untrained items on the translation recognition task for the three morphological conditions. Error bars indicate standard errors. Asterisks denote significant effects in paired comparisons of the morphological conditions (*p* < 0.05).

For trained items, we found a significant main effect of subregion (see Table 4), showing greater activation in anterior STG (aSTG) compared to posterior STG (*p* < 0.001). There was also a main effect of condition, revealing greater activation for the complex non-linear condition compared to the complex linear condition (*p* < 0.001), and for the simple compared to the complex linear condition (*p* < 0.001; see Figure 5). For untrained items, we found a main effect of condition (see Table 4), showing greater activity for the simple compared to the complex linear condition (*p* = 0.008; see Figure 5).

##### Middle temporal gyrus.

**Figure 6.**
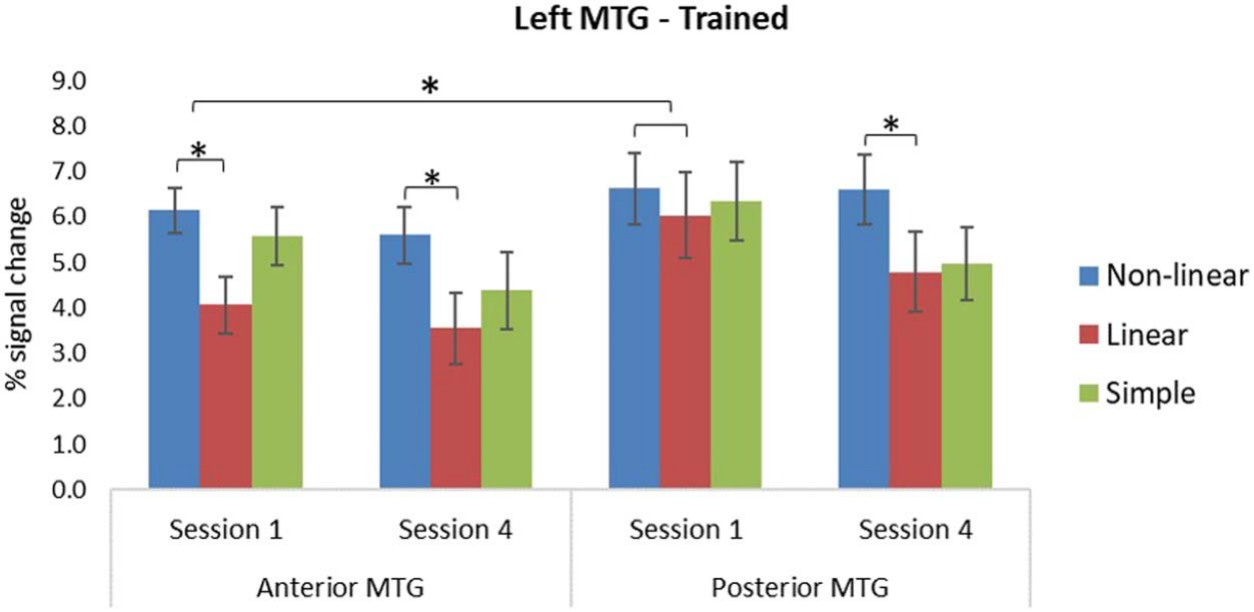
Percent signal change in anterior and posterior subregions of the left middle temporal gyrus (MTG) for trained items on the translation recognition task for the three morphological conditions split by the first and the last sessions. Error bars indicate standard errors. Asterisks denote significant effects in paired comparisons of the morphological conditions (*p* < 0.05).

**Figure 7.**
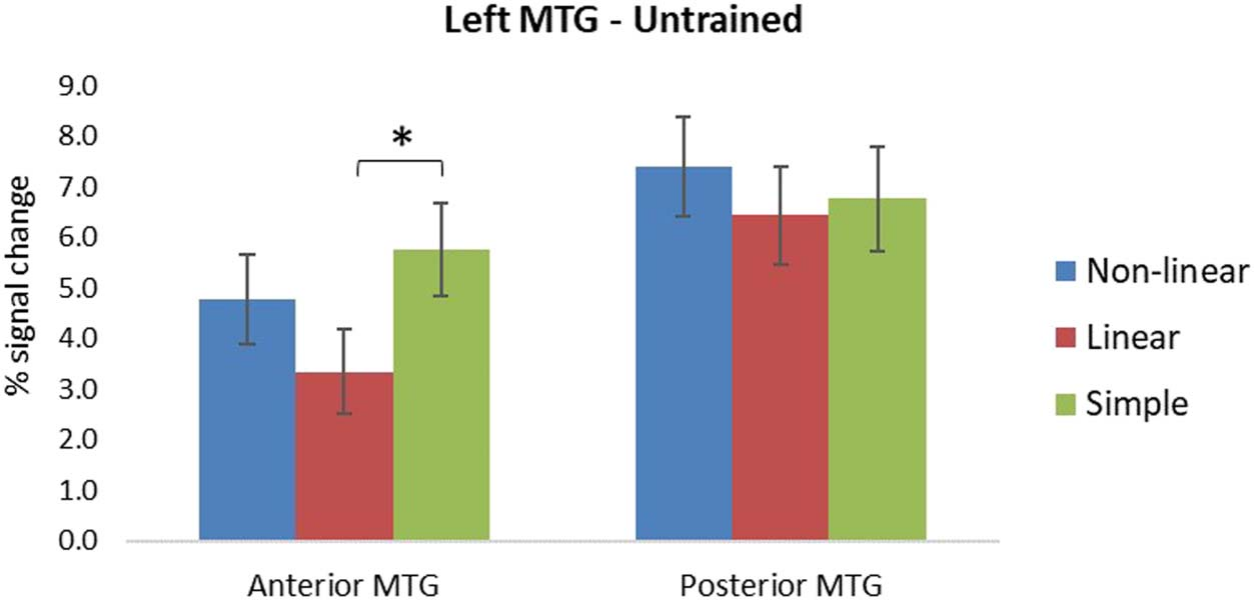
Percent signal change in anterior and posterior subregions of the left middle temporal gyrus (MTG) for untrained items on the translation recognition task, for the three morphological conditions. Error bars indicate standard errors. Asterisks denote significant effects in paired comparisons of the morphological conditions (*p* < 0.05).

For trained items, there was a main effect of condition, with the complex non-linear condition showing greater activation in comparison to the complex linear condition: (*p* = 0.008). There was an interaction of condition and subregion, and a marginal three-way interaction of condition, subregion, and session, indicating that the difference in activation between the complex non-linear and linear condition was greater in anterior MTG compared to posterior MTG only in the first session, *F*(1, 27) = 5.62, *p* = 0.025, *η*_*p*_^2^ = 0.172 (see Figure 6). For untrained items, there was a main effect of subregion (see Table 4), showing greater activation in posterior MTG than anterior MTG (*p* = 0.009). There was also an interaction of condition by subregion, which revealed greater activation for the simple condition compared to the complex linear condition only in anterior MTG, *F*(1, 27) = 10.81, *p* = 0.003, *η*_*p*_^2^ = 0.286 (see Figure 7).

##### Hippocampus.

For trained items, there was a significant main effect of subregion, revealing greater activation in posterior compared to anterior hippocampus (*p* = 0.002), and no effect of condition. There were no significant effects in the hippocampus for untrained items.

#### Brain-behaviour correlations

##### Measures of performance in the experimental task.

Our planned correlational analyses showed that these measures, computed on the translation selection task outside the scanner, that is, total learning (S4T2–S1T1), consolidation (S2T1–S1T2), and performance on untrained words (S4 UT), were not correlated with changes in brain activation from the first to the last session in any condition.

##### Prior linguistic abilities.

**Figure 8.**
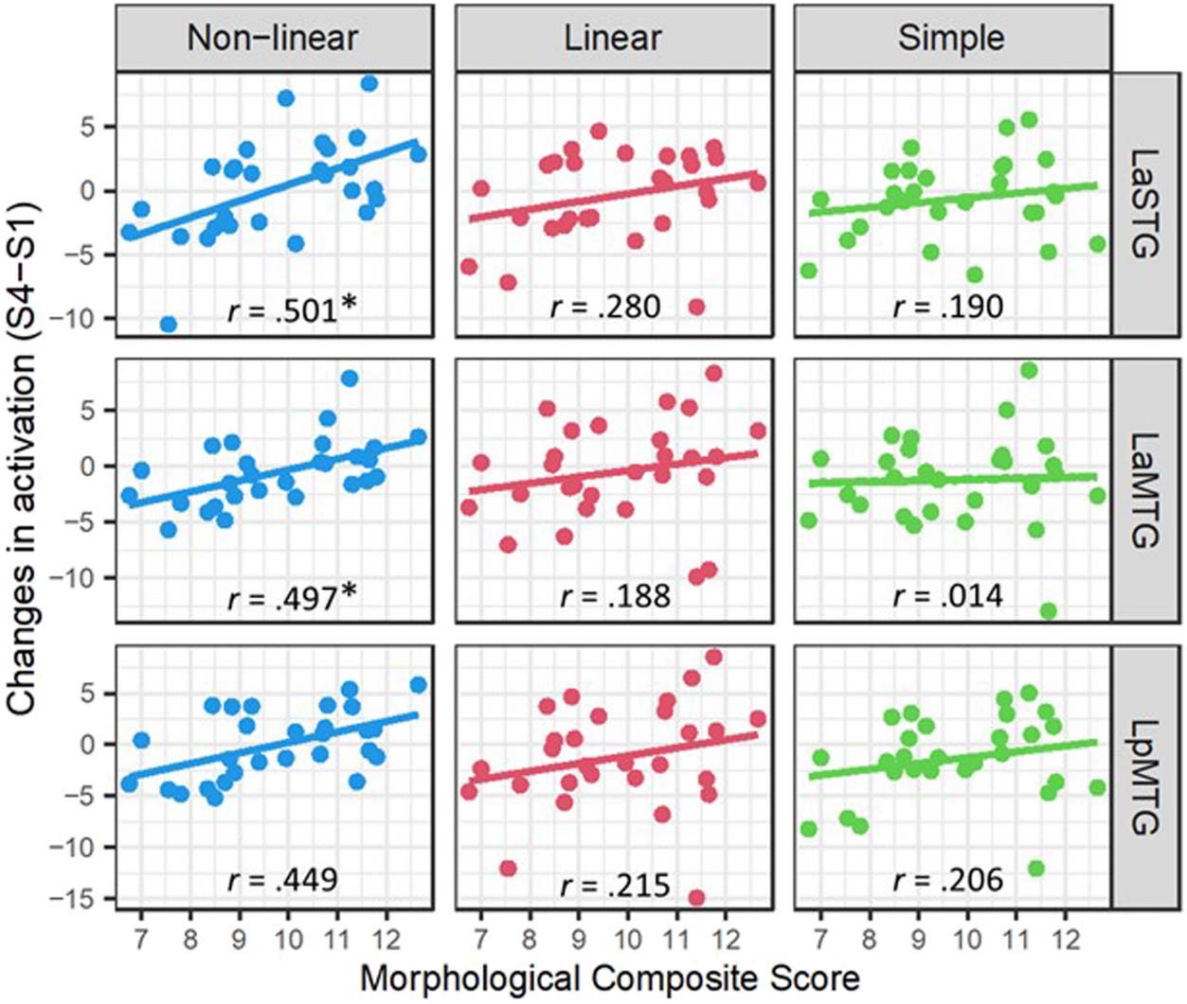
Correlation between participants’ morphological awareness in L1 and activation changes from first to last session (S4–S1) in temporal regions of interest: left anterior superior temporal gyrus (LaSTG), left anterior and posterior middle temporal gyrus (LaMTG and LpMTG). Asterisks denote significant effects after the Bonferroni correction for multiple comparisons.

The morphological composite score showed a positive significant correlation with changes in brain activation from the first to the last session only for the complex non-linear condition, in left aSTG (*r* = 0.501, *p* = 0.007) and left anterior MTG (*r* = 0.497, *p* = 0.007; see Figure 8). This suggests that individuals with high morphological awareness in L1 showed a greater increase in activity in these regions during training for the non-linear condition. This effect was also significant in posterior MTG (*r* = 0.449, *p* = 0.016) but did not survive the Bonferroni correction for multiple comparisons. No correlations were found for other conditions, or in other ROIs. No correlation was found for the phonological composite score. Full results from the correlational analysis with the morphological and phonological composite scores are presented in the Supporting Information (see Table S1).

### Exploratory Analyses

We conducted a set of exploratory correlations to further examine the effect of behavioural performance on brain activity, the effect of STM on brain activity, and correlations of hippocampus and neocortical activations.

#### Correlations with behavioural performance

Because the analysis of the behavioural task performed outside the scanner showed a significant interaction between session and test (for accuracy), and between session, test, and condition (for RT), with greater improvement during the first session, at least in some conditions, we were interested in focusing on brain activation that reflected learning within the first session. We therefore tested the correlation between brain activation in the first session (S1) with improvement during the first training session outside the scanner on the 4-AFC translation selection task. Within-S1 improvement was measured as the difference between the tests conducted before and after the first training session (S1T2–S1T1), separately for each condition.

**Figure 9.**
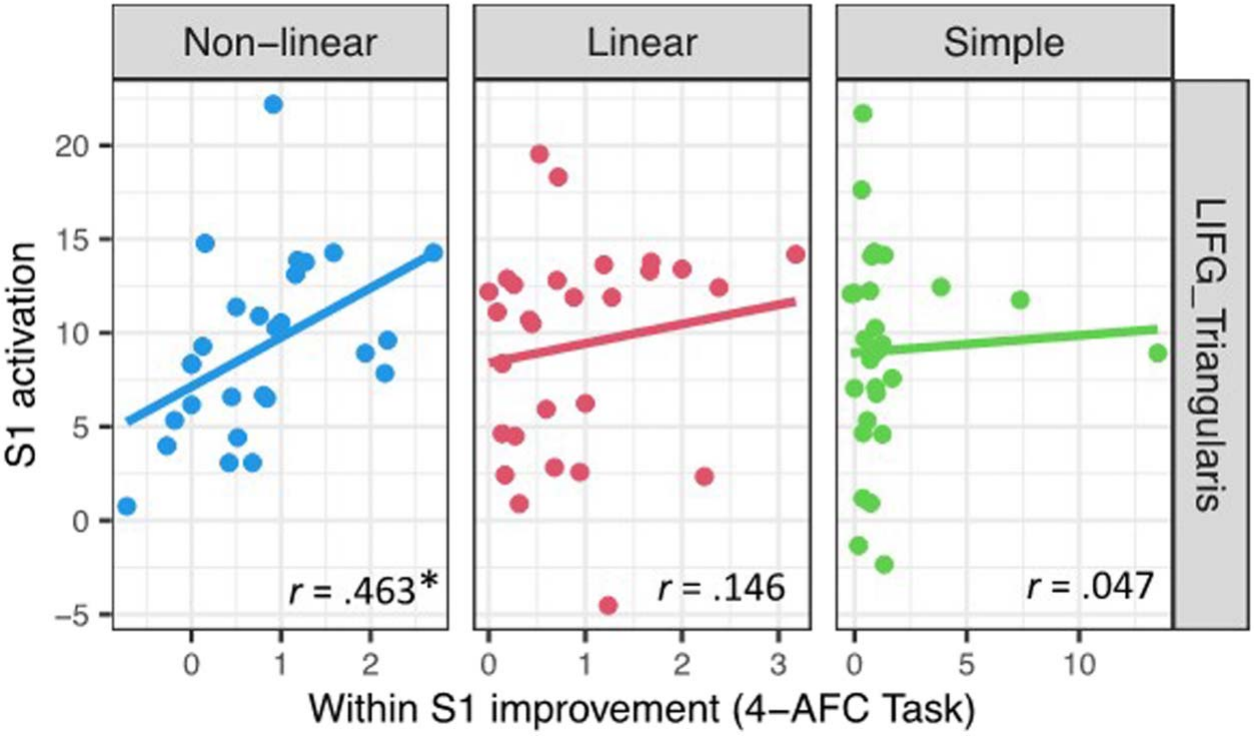
Correlations between activation in Session 1 and Session 1 improvement on the 4-alternative forced choice (4-AFC) translation selection task, in left inferior frontal gyrus (LIFG) pars triangularis, including outliers. Asterisks denote significant effects after the Bonferroni correction for multiple comparisons.

Only for the complex non-linear condition, improvement within Session 1 was significantly correlated with activation in Session 1 in LIFG pars triangularis (see Figure 9). The results remained significant after removing an outlier that was 2.5 *SD*s above the mean of activation in session 1 (*r* = 0.537, *p* = 0.005; see Figure 9).

#### Correlations with short-term memory

While working memory and STM have been found to be correlated with behavioural (Gray et al., 2022; Gupta et al., 2003) and neural (Ylinen et al., 2020) measures of word learning in general, a more specific role in learning inflectional morphology (Williams & Lovatt, 2003) and irregular nouns (Kempe & Brooks, 2008) has also been shown. We therefore tested the correlation between participants’ STM, assessed using the forward digit span task, and brain activation in each condition, averaged across sessions. A Bonferroni correction was applied to account for multiple comparisons for the three morphological conditions for each test and the number of subregions in each ROI.

We found significant positive correlations of digit span forward with average brain activation in LIFG triangularis for all three conditions, but these survived the Bonferroni correction only for the complex non-linear condition, *r* = 0.505, *p* = 0.006 (complex linear, *r* = 0.501, *p* = 0.007; simple, *r* = 0.479, *p* = 0.010). There was also a correlation with average activation in LIFG opercularis for the complex non-linear condition that did not survive the Bonferroni correction, *r* = 0.478, *p* = 0.010.

#### Correlations between hippocampal and cortical regions

Finally, to test the predictions of the CLS model for a general shift in reliance on neural structures during leaning, we were also interested in examining relations between activation changes throughout the learning process in the hippocampal and cortical regions. We therefore tested correlations between the first to last session (S4–S1) changes in activation in anterior and posterior hippocampus and the first to last session changes in cortical ROIs, separately for each morphological condition (see Table 5). In contrast to the hypothesis, increase in hippocampal activity from the first to the last session positively correlated with an increase in activation in all cortical regions for the complex linear condition, this was particularly evident in the posterior portion of the hippocampus after correcting for multiple comparisons for the three morphological conditions and the number of subregions in each cortical ROI, and 2 subregions of the hippocampus: LIFG (3 subregions × 3 conditions × 2 hippocampal areas, *p* = 0.05/18 = 0.003), STG (2 subregions × 3 conditions × 2 hippocampal areas, *p* = 0.05/12 = 0.004) and MTG (2 subregions × 3 conditions × 2 hippocampal areas, *p* = 0.05/12 = 0.004).

**Table 5.** Correlations of first to last session (S4–S1) changes in activation in anterior and posterior hippocampus with cortical regions of interest for the three morphological conditions

	Left anterior hippocampus			Left posterior hippocampus		
	Non-linear	Linear	Simple	Non-linear	Linear	Simple
Left anterior STG	0.372	0.402*	0.371	0.045	0.614**	0.338
Left posterior STG	0.197	0.437*	0.189	−0.025	0.594**	0.070
Left anterior MTG	0.667**	0.519**	0.406*	0.186	0.578**	0.211
Left posterior MTG	0.275	0.483*	0.401*	−0.086	0.645**	0.172
LIFG pars Opercularis	0.256	0.375*	0.289	−0.289	0.526**	0.161
LIFG pars Orbitalis	0.034	0.416*	0.354	−0.230	0.621**	0.264
LIFG pars Triangularis	0.097	0.330	0.274	−0.339	0.419*	0.182

*Note*. STG = superior temporal gyrus, MTG = middle temporal gyrus, LIFG = left inferior frontal gyrus. *Significant without Bonferroni correction; **significant with Bonferroni correction.

### Sensitivity Analysis

We conducted a sensitivity analysis using G*power 3.1 (Faul et al., 2007), using our current sample size of 28, α = 0.05, and a power of 80%, separately for the three-way and two-way repeated measures ANOVA including within-subject interactions, and for the brain-behaviour correlations. The sensitivity analysis revealed that our current sample size had enough power to detect an effect size of *f* ≥ 0.246 (i.e., *η*_*p*_^2^ ≥ 0.057) for the three-way ANOVA, and an effect size of *f* ≥ 0.275 (i.e., *η*_*p*_^2^ ≥ 0.070) for the two-way ANOVA. All significant interactions in the current study showed larger effect sizes. For brain-behaviour correlations, sensitivity analysis with the above parameters revealed that our sample size can detect *r* ≥ 0.451. All our significant correlations are larger. These results suggest that any real effects of a smaller size may have been missed.

## DISCUSSION

The current preregistered study examined neural mechanisms associated with the learning and generalization of morphologically derived words in an artificial morpho-lexicon. Across four sessions, participants were trained on two morphologically complex conditions, constructed using linear and non-linear derivations, and a third control condition of morphologically simple words (used as a baseline). Results from the preregistered analyses of the fMRI data are discussed below along with the behavioural and exploratory analyses, based on our four key preregistered research questions. We focus here on the neural findings, which are the main contribution of the current study. Further discussion and analysis of the behavioural findings can be found in our previous paper (Nathaniel et al., 2023).

Our behavioural results from both tasks performed inside and outside the scanner show clear improvement in accuracy and reaction time across the four sessions, indicative of learning. While performance inside the scanner during the first scan did not exceed chance level, this task occurred early in learning and immediately after performance outside the scanner was clearly above chance in all conditions. Given the correlations found between performance in and out of the scanner, these results suggest that even during the first scan participants had already acquired some knowledge of word meanings, which may not be reflected in the translation recognition task, performed inside the scanner, because it was more difficult. (For more discussion of the differences between tasks, see Figure S1 in the Supporting Information.)

### Question 1: Are Morphologically Derived Complex Words Decomposed in Early Stages of Word Learning?

Our behavioural results (both inside and outside the scanner) showed better generalization of both the complex linear and non-linear conditions in comparison to the simple condition measured at the last training session, providing strong behavioural evidence for decomposition of derived words at that time point (Nathaniel et al., 2023). Neurally, we predicted that trained words would show greater activation in LIFG for the morphologically complex conditions in comparison to the simple condition across both sessions (S1 and S4). Our planned fMRI analysis did not reveal a significant difference between the morphological conditions in LIFG. However, our exploratory analyses examining brain-behaviour correlations showed that behavioural improvement during training within the first training session (T2–T1 in S1) was correlated with activation in LIFG pars triangularis in S1, only for the complex non-linear condition (Figure 8). These results are partially in line with our hypothesis as they show the involvement of LIFG pars triangularis, which has previously been associated with decomposition (Bitan et al., 2020; Bozic et al., 2007; Nevat et al., 2017), in the early stage of processing morphologically complex trained words. In contrast to our hypothesis, this was only evident in the complex non-linear condition, and not in the linear condition, as will be discussed below.

There is evidence for the involvement of LIFG pars triangularis (Bitan et al., 2020; Bozic et al., 2007; Marangolo et al., 2006) in the processing of morphologically derived words in L1, and in proficient L2 speakers (Bick et al., 2011; De Grauwe et al., 2014). Given the role of left pars triangularis in lexical retrieval (Nevat et al., 2017; Ullman, 2004, 2016), its involvement in decomposition of derived words may reflect the access and retrieval of the meaning of derivational morphemes. The current results demonstrate reliance on LIFG pars triangularis in the very early stages of learning morphologically derived words, suggesting that decomposition can occur as early as the first training session.

Our results for trained words show this reliance on dorsal LIFG only for the complex non-linear words, while the complex-linear condition did not show similar results. Previous studies showed the involvement of dorsal LIFG also in processing of linearly derived words (Bozic et al., 2007; Marangolo et al., 2006; Meinzer et al., 2009; Pliatsikas, Wheeldon, et al., 2014). The absence of correlation between performance in the complex linear condition and activation in LIFG in the current study cannot be attributed to absence of decomposition in this condition. Untrained words with a linear structure showed higher accuracy than simple words, suggesting that the monosyllabic roots and suffix morphemes of the linear structures were indeed decomposed. Moreover, performance on the 4-AFC translation selection task for trained words with a linear structure was more accurate than in the other two conditions, suggesting that decomposition of words in the linear condition occurred even during training.

An alternative interpretation for these results is that decomposition of the linear structure was more transparent and thus easier than that of the non-linear condition, due to its perceptual saliency (Schwarzwald, 2002). Words sharing roots or suffixes in the linear condition share full syllables, which may be easier to detect, while detecting shared root morphemes or templates in the non-linear condition requires sub-syllabic segmentation. This interpretation is supported by behavioural findings from statistical learning paradigms, which require parsing of speech streams into words, and showed that non-adjacent dependencies are harder to learn compared to adjacent dependencies (Saffran et al., 1997; Sandoval & Gómez, 2013). In the current study, the relative ease of parsing the linear structure may be the reason that this did not require increased reliance on LIFG. Previous studies have shown greater reliance on LIFG pars opercularis in visual-spatial sequences with non-adjacent compared to adjacent dependencies (Bahlmann et al., 2009). These findings may explain the reliance of performance in the non-linear condition but not in the linear condition on activation in LIFG, despite behavioural evidence of decomposition in both conditions. It is also pertinent to consider potential implications of our findings for speakers of languages with a more concatenative profile, such as English. These speakers may show a larger difference in morphological decomposition between linear and non-linear complex words, because decomposition of the linear formation would not only be more transparent but also more familiar than the non-linear formation, which is infrequent in English.

### Question 2: Does Processing of Untrained Morphologically Derived Complex Words Involve More Decomposition, and Faster Creation of Lexical Representations Compared to Simple Words?

As indicated above, our behavioural results for the untrained words on both tasks (translation recognition and 4-AFC translation selection) showed more accurate performance for morphologically complex words compared to simple words, indicating better generalization. As for the fMRI results for the untrained words, we predicted greater activation for the complex conditions in comparison to the simple condition in dorsal LIFG, associated with decomposition. Our planned analyses were partially in line with this hypothesis, showing greater activation in the non-linear compared to the simple condition in LIFG pars triangularis and pars opercularis. These results are consistent with our findings for trained words, and with the implication of dorsal LIFG in morphological decomposition (Bick et al., 2010; Bitan et al., 2020), and suggest that after our participants had learned the non-linear structure, they decomposed these complex words, even during the brief exposure to the untrained words. However, similar to the trained words, there was no difference in LIFG between the complex linear and simple condition.

We also predicted that untrained words with a complex morphological structure would show greater activation than simple words in temporal regions associated with the formation of whole-word lexical representations, due to the facilitating effect of the familiar morphemes. This prediction was not supported. In contrast, untrained words in the simple condition elicited *more* activation than complex words (with a significant difference from the linear condition) in left STG and in left anterior MTG. These temporal regions have been associated with activation of lexical phonological and lexical semantic representations, respectively (Binder et al., 2000; Bozic et al., 2013; Tyler et al., 2005; Whiting et al., 2015), and were found in early stages of learning novel words (Davis et al., 2009; Mårtensson et al., 2012). Given the lower accuracy of simple compared to complex untrained words, both inside and outside the scanner, we cannot interpret this higher activation as better formation of lexical representation but rather as potentially reflecting participants’ efforts to form item-specific lexical representations of these untrained words.

### Question 3: Are Morphologically Derived Complex Words Learned Better and Consolidated Faster Than Simple Words?

Our predictions were based on the hypothesis that morphologically decomposable words would be faster to learn and faster to form lexical representations. Based on the assumptions of the CLS model, we predicted that learning and consolidation of new words would involve increased reliance on cortical areas accompanied by decreased reliance on hippocampal activation (Davis & Gaskell, 2009; McClelland et al., 1995).

#### Learning

Our behavioural results from the 4-AFC translation selection task showed higher overall accuracy for trained words from the complex linear condition compared to the simple and complex non-linear conditions, suggesting that the decomposition of the linear morphological structure facilitated the learning of the meaning of trained words. Note that while we found higher overall accuracy for the complex linear condition in the translation selection task, in contrast to our hypotheses we did not find differences between conditions in the amount of improvement (total learning from the first test in the first session to the last test in the last session). These results suggest that the advantage of morphological decomposition for learning the meaning of new words is evident very early on and is eliminated by the end of the fourth session of training on the same words.

Our prediction for the fMRI results that changes in activation in the fronto-temporal ROIs from the first to the last session in the complex conditions would correlate with overall behavioural improvement (i.e., total learning), was not supported for any of the conditions. We also hypothesized that complex words would show a greater first to last session increase in activation in frontal and temporal regions compared to the simple condition, evident in an interaction of session and condition. Our planned ROI analysis revealed only a three-way interaction of session, condition, and subregion in left MTG, which was not in the predicted direction.

Our planned ROI analysis for trained words did reveal a main effect of condition in temporal regions. The findings showed greater activation in left STG for the non-linear and simple conditions compared to the linear condition. This effect of condition is also shown in the whole-brain analysis as greater activation in the simple compared to the linear condition in bilateral STG. These findings are in line with behavioural results in the translation selection task, showing higher accuracy in the complex linear condition compared to the other two, suggesting that because the other conditions were more difficult, they generated more activation in regions related to the formation of new lexical phonological representations (Veroude et al., 2010). The ROI analysis also showed greater activation in left MTG, associated with activation of lexical-semantic representations (Binder et al., 2009; Yang et al., 2015), for the non-linear compared to the other two conditions throughout training. Given that trained words in the complex non-linear condition were also the only words for which performance was correlated with activation in dorsal LIFG, these findings may suggest that the effortful decomposition of complex non-linear words enhanced the lexical-semantic processes.

These results are in line with previous studies examining morphological processing in L1, showing greater dependence of derived words than morphologically simple words on left temporal areas, including STG and MTG (Vannest et al., 2011), and specifically anterior temporal regions (Bölte et al., 2010) involved in morpho-semantic processing. There is also evidence from MEG studies showing early activation of morphologically complex words in temporal regions in the early stages of word reading (Fruchter & Marantz, 2015; Solomyak & Marantz, 2009). Our results extend these effects to the processing of novel spoken words.

#### Consolidation

We predicted faster consolidation and more offline improvement in the morphologically complex conditions. Based on the assumption of the CLS model that increased reliance on cortical areas would be accompanied by *decreased* reliance on hippocampal activation (Davis & Gaskell, 2009; McClelland et al., 1995) we predicted that complex words would show less hippocampal activation in S1 compared to the simple condition, and that the simple condition would show a decrease in hippocampal activation from S1 to S4. In contrast to these predictions, we did not find differences between conditions in behavioural measures of offline consolidation after the first session. We also did not find any condition-by-session interactions in the hippocampus. These results suggest that although morphological decomposition facilitates the learning of the meaning of new words, this effect was not driven by differences in consolidation.

Nevertheless, our exploratory analysis revealed findings associating activity in the hippocampus with the complex linear condition. We found a *positive* correlation between increase in activation from S1 to S4 in the left posterior hippocampus from the first to the last session and increase in cortical activation only for the complex-linear condition (see Table 5). The complex-linear condition was the easiest to decompose and the fastest to learn, as shown by our behavioural results. The anterior hippocampus has been associated with the encoding of novel stimuli (Dolan & Fletcher, 1997; Strange et al., 1999) and in pattern completion (Poppenk et al., 2013), while the posterior hippocampus has been associated with retrieval of episodic memory representations (Nadel & Moscovitch, 1997) and with pattern separation (Grady, 2020; Poppenk et al., 2013). The hippocampus has been shown to be involved in inferring the meaning of separate stimuli following the learning of compounds. A study with rats that learned the value of compound stimuli (AB), showed that lesions to the hippocampus prevented the rats from inferring the value of the constituent stimuli (A, B), and that the critical stage for the integrity of the hippocampus was the retrieval (Gilboa et al., 2019). This may suggest that the involvement of the hippocampus in learning complex linear words is related to inferring and retrieving the meaning of individual morphemes following the learning of whole words.

An alternative interpretation for the involvement of the hippocampus in learning the complex-linear words may be its involvement in pattern separation and in reducing interference between overlapping memories during retrieval (Favila et al., 2016; Kesner, 2013). As noted above, words that share morphemes in our complex-linear condition were more similar to each other, and may be harder to tell apart, than words sharing morphemes in the complex non-linear condition, because the shared morphemes in the complex-linear condition are full syllables. Behavioral studies show the critical role of the first syllable in word recognition (Allopenna et al., 1998). Thus, the involvement of the hippocampus in the complex linear condition may reflect the effort to avoid interference between related words during retrieval.

It is also worth noting that the positive correlation found between the changes in the hippocampus and cortical regions over the course of training stands in contrast to the predictions of the CLS and other standard system consolidation theories (e.g., Dudai, 2012), which suggest that the dynamics of learning and consolidation involves a shift from reliance on hippocampal activity to increased reliance on cortical areas (Davis & Gaskell, 2009). Several studies found evidence for the involvement of the hippocampus in initial stages of novel word learning (Breitenstein et al., 2005; Davis & Gaskell, 2009). However, there is no direct evidence for a shift from hippocampus to cortical regions after consolidation of newly learnt words (Tartaro et al., 2021), nor are there differences in activation in the hippocampus between novel and trained words (Landi et al., 2018).

One possible explanation for the unexpected positive correlation between hippocampal and neocortical changes is that while most theoretical accounts and empirical studies of consolidation focus on a single encoding event, which could be followed by several testing points, the current study used multisession training, which better reflects the learning of natural languages. Thus, the repeated encoding of the words may have resulted in the positive correlation between hippocampal and neocortical learning related changes. Another possible interpretation is that this positive correlation is more consistent with recent approaches to memory formation, such as trace transformation theory (Sekeres et al., 2018; Winocur et al., 2010; Winocur & Moscovitch, 2011) and Neural-Psychological Representation Correspondence (Gilboa & Moscovitch, 2021). These theories suggest that multiple neural and mental representations are formed during and immediately after an event, in hippocampal and cortical areas, including detailed episodic representations as well as more abstract schematic and semantic ones. According to this approach these multiple representations co-exist and undergo interactive, dynamic changes in strength, composition, and dominance of expression. This theory is supported by findings from autobiographical memories showing the same level of activation in posterior hippocampus even after two years (Bonnici & Maguire, 2018).

### Question 4: Is There an Effect of Participants’ Prior Linguistic Abilities in L1 on the Neural Processes Involved in Learning of Newly Learned Derived Words?

While we expected both morphological and phonological abilities to correlate with first to last session changes in activation in our ROIs for the complex conditions, our planned analysis revealed such a correlation only for morphological abilities and only for the complex non-linear condition. Specifically, increase in activation in temporal areas, from the first to the last session for trained words in the complex non-linear condition was positively correlated with participants’ morphological composite score measured in their L1. This score reflects participants’ ability to process Hebrew roots (the morphological relations test) and templates (the morphological fluency test), which have a non-linear structure. This may explain why the correlation was found specifically for the complex non-linear condition, which is the most similar to the morphological roots and templates in Hebrew, and not for the complex linear condition. The finding of this correlation in superior and middle temporal gyri, which have been associated with access to phonological and lexico-semantic representations of both derived and simple words (Bölte et al., 2010; Cavalli et al., 2016; Vannest et al., 2011), suggests that morphological processing facilitates the formation of lexical representations during training. Altogether these results support the conclusion that better morphological abilities in L1 are associated with greater neural plasticity in forming lexical representations and linking them to new words with a similar morphological structure. Another possible interpretation for the lack of any correlation in the complex linear condition could be the relative ease and salience of the morphological structure in this condition, as mentioned in our discussion of our findings related to Question 1. Nonetheless, further research exploring the neural mechanisms underlying morphological processing in languages with a more concatenative profile could provide valuable insights into the universality and variability of prior linguistic abilities in L1 contributing to word learning across languages.

### Correlations With Short-Term Memory

Lastly, in our exploratory analysis we also examined the effect of participants’ STM, reflected via the forward digit span task, on brain activation in our ROIs, for each condition, averaged across sessions. This analysis revealed significant correlations between STM and activation in LIFG pars triangularis across all conditions, but survived Bonferroni correction only for the complex non-linear condition. This finding is consistent with the well-known role of LIFG triangularis in working memory and STM (Nee & Jonides, 2011; Rogalsky et al., 2008; van Ewijk et al., 2015; Xue et al., 2004) and with our behavioural results (Nathaniel et al., 2023) showing a positive correlation of performance on the forward digit span task with average performance outside the scanner on untrained words, across all conditions. These results show the important role of STM in novel word learning, also shown in previous behavioural (e.g., Dracos, 2013; Jarrold et al., 2009) and neuroimaging (Ylinen et al., 2020) studies.

### Limitations

The current study used regular, transparent morphology and a blocked order of training of the different morphological conditions to facilitate morphological learning. To measure morphemic knowledge for each of the two morphemes comprising a word, all trials contained a distractor with a shared morpheme alongside the correct response. These design features, along with including more complex than simple words, could have increased the participants’ sensitivity to the morphological structure of words. It is possible that using irregular and exception words, a random training order, or a smaller proportion of complex words could make morphological decomposition harder to deploy, especially in early stages of learning. While our planned analyses supported only some of our preregistered predictions, additional support was provided by the exploratory analyses. Therefore, one of the main limitations of this study is the lack of confirmatory evidence for many of our findings. Another limitation of the study is its sample size (*N* = 31), selected in light of its very demanding five session protocol. While the sensitivity analysis showed that it was large enough to provide enough power for the effect sizes we found, any real effects that are smaller were probably missed in the current study. Lastly, our participants had considerable proficiency in additional languages, and especially English. This may have contributed to their word learning ability in general, as there is evidence suggesting an advantage for multilinguals in learning novel words (Hirosh & Degani, 2018).

## CONCLUSIONS

In summary, our results provide novel insights into the neural mechanisms underlying the learning and decomposition of morphologically derived words in a novel morpho-lexicon. While our behavioural results showed decomposition of derived words with both linear and non-linear structures in the early stages of learning, fMRI results suggest that different neural mechanisms underlie learning of these two types of complex words. Learning and generalization of words with a non-linear structure was shown to rely more on left frontal regions, typically associated with morphological decomposition. Individuals’ ability to process the non-linear morphology in their L1, Hebrew, was related to their increase in activation in temporal areas during learning, presumably due to the quick formation of lexical representations for the new words. These results lend support for the conclusion that decomposition of derived words can occur in early stages of learning, and also suggests that there is transfer of morphological abilities in participants’ L1 to the learning of novel artificial morpho-lexicon. They further suggest that morphological decomposition can enhance the formation of lexical-semantic representations.

In contrast, derived words with a linear structure seem to be easier to decompose, evident by higher accuracy during training. This may be explained by their more perceptually salient structure in which full syllables are preserved across morphologically related words. Words with a linear structure showed the least activation in temporal areas for both trained and untrained words. Interestingly, processing words with a linear structure relied more heavily on activity in the anterior and posterior parts of the hippocampus. This may reflect either inferential processes in determining the meaning of parts of whole words, or pattern separation processes involved in the interference between morphologically related words.

Lastly, while our predictions were planned in accordance with the CLS model, we found no support for a shift from reliance on hippocampal to reliance on cortical areas in any of the conditions. In contrast, our finding of a positive correlation between the changes in hippocampus and cortical areas are more in line with recent theories suggesting that representations in these regions co-exist and continue to interact with one another beyond initial learning.

## ACKNOWLEDGMENTS

We thank Dan Manor for his help with programming and setting up the fMRI tasks. We are also grateful to two anonymous reviewers for their helpful suggestions and comments.

## FUNDING INFORMATION

Vedran Dronjic, National Science Foundation (https://dx.doi.org/10.13039/100000001), Award ID: BCS 1753611. James R. Booth, National Science Foundation (https://dx.doi.org/10.13039/100000001), Award ID: BCS 1753626. Tali Bitan, United States–Israel Binational Science Foundation (https://dx.doi.org/10.13039/100006221), Award ID: 2017613.

## AUTHOR CONTRIBUTIONS

**Upasana Nathaniel**: Data curation; Formal analysis; Investigation; Methodology; Project administration; Visualization; Writing – original draft; Writing – review & editing. **Stav Eidelsztein**: Investigation; Project administration. **Kate Girsh Geskin**: Investigation; Project administration. **Brianna L. Yamasaki**: Methodology; Writing – review & editing. **Bracha Nir**: Conceptualization; Funding acquisition; Writing – review & editing. **Vedran Dronjic**: Conceptualization; Funding acquisition; Writing – review & editing. **James R. Booth**: Conceptualization; Funding acquisition; Writing – review & editing. **Tali Bitan**: Conceptualization; Funding acquisition; Supervision; Writing – review & editing.

## DATA AND CODE AVAILABILITY STATEMENT

All code and data are available at https://osf.io/ju7vh/.

## Supplementary Material



## TECHNICAL TERMS

Decomposition: Involves breaking down complex words into smaller units to understand their meaning and structure.
Morphemes: Minimal units of words that have meaning and cannot be subdivided further.
Consolidation: Process by which new and initially unstable memories transform into long-term memories.
Generalization: Application of knowledge learned in one context to new, similar situations.
Derivational morphology: Involves forming new words by adding prefixes or suffixes to existing words to change their meaning or grammatical category.
