# Supplementary material for: Neural Mechanisms of Learning and Consolidation of Morphologically Derived Words in a Novel Language: Evidence From Hebrew Speakers
Source: Neurobiol Lang (Camb). 2024 Sep 11;5(4):864–900. doi: 10.1162/nol_a_00150 (PMC11410356; doi:10.1162/nol_a_00150)
Supplement: Supplementary file 1 [file nol-5-4-864-s001.pdf]

Supplementary Materials

Due to the discrepancy between the translation selection and translation recognition tasks, we conducted further analysis of the translation recognition task, taking into account the type of presented translation: correct or incorrect (it should be noted that all incorrect translations share a morpheme with the correct translation, and the corresponding manipulation was also done for the simple condition). We ran a repeated-measures ANOVA for participants' average accuracy on the task, with *session* (S1 vs. S4), *morphological condition* (complex non-linear, complex linear, simple), and *translation type* (correct vs. incorrect) as within subject factors. We found a main effect of session: $F(1,27) = 86.86$, $p < .001$, condition: $F(2,54) = 3.78$, $p = .029$, and translation type: $F(1,27) = 20.93$, $p < .001$, as well as a significant interaction between condition and translation type: $F(2,54) = 16.76$, $p < .001$. A follow-up analysis split by translation type revealed a significant main effect of condition only for trials with incorrect translations: $F(2,54) = 19.62$, $p < .001$, showing significantly higher accuracy on the simple condition in comparison to both the complex non-linear: $t(27) = 6.42$, $p < .001$, and complex linear conditions: $t(27) = 5.16$, $p < .001$ (see figure S1 in Supplementary Materials). The difference between the two complex conditions was not significant: $t(27) = .039$, $p = .969$. There was no main effect of condition for trials with correct translations: $F(2,54) = 2.07$, $p = .136$.

Keeping in mind that all incorrect translations shared a morpheme with the correct translation, these results suggest that, in the translation recognition task, it was harder to reject incorrect translations of morphologically complex words because these distractors shared a morpheme with the correct translation. The incorrect translation with a shared morpheme is less distracting in the 4-alternative forced choice translation selection task, where they appear alongside the correct translation. These results may suggest that the translation recognition task was more difficult than the translation selection task, and may explain the overall low accuracy levels in the translation recognition task (performed inside the scanner). These results may also explain the discrepancy between tasks in the effect of condition. Overall, these results show that the ability to decompose complex words contributes to word learning as it facilitates matching a word with its translation when the correct translation is presented, although it may interfere when a learnt morpheme is present in an incorrect translation.

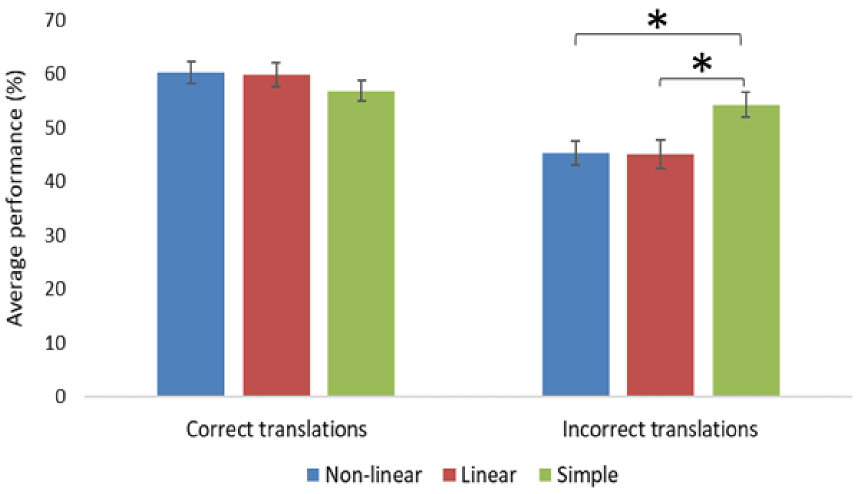

Figure S1. Mean percentage accuracy on the Translation Selection task, for correct and incorrect translation types, split by performance for each morphological condition, only for trained items. Error bars indicate standard errors. Asterisks denote significant effects (*p* < .05).

Table S1: Brain-behavior correlation of activation changes from first to last session (S4 – S1) with participants' phonological and morphological composite score.

	**Phonological Composite Score**			**Morphological Composite Score**		
	Non-linear	Linear	Simple	Non-linear	Linear	Simple
Left anterior STG	.032	-.089	-.349	.501**	.280	.190
Left posterior STG	.033	-.022	-.295	.325	.300	.137
Left anterior MTG	.179	.084	-.095	.497**	.188	.014
Left posterior MTG	.231	.093	-.177	.449	.215	.206
LIFG pars Opercularis	.054	-.033	-.376	.219	.044	.045
LIFG pars Orbitalis	.034	.011	-.228	.087	.116	-.184
LIFG pars Triangularis	-.038	-.089	-.290	.124	-.020	-.095
Left anterior Hippocampus	.102	.186	.163	.165	.091	-.060
Left posterior Hippocampus	.089	.288	.161	-.136	.054	-.120

**Significant after Bonferroni correction
